# Application of *Panax notoginseng* Saponins in Skin Wound Repair: Mechanisms and Research Progress

**DOI:** 10.1111/jcmm.71381

**Published:** 2026-10-14

**Authors:** Zhifeng Wang, Ting Shi, Daman Tian, Rui Li, Ting Yao, Junfeng Lan, Yujiang Xi, RuiRui Wang, Jian Wang

**Affiliations:** ^1^ The First Clinical Medical College Yunnan University of Chinese Medicine Kunming China; ^2^ College of Chinese Materia Medica Yunnan University of Chinese Medicine Kunming China

**Keywords:** clinical applications, dosage form innovation, *Panax notoginseng* saponins (PNS), signalling pathways, skin wound repair

## Abstract

Skin wound repair involves four stages: haemostasis, inflammation, proliferation and remodelling. Refractory wounds feature prolonged healing cycles, severe scar hyperplasia and limited clinical therapeutic strategies. *Panax notoginseng* saponins (PNS), primarily the ginsenosides Rg1 and Rb1 monomers, display high safety and multitarget regulatory properties across the entire wound repair cycle. PNS modulates the inflammatory microenvironment and induces M2‐type polarization of macrophages by inhibiting NF‐κB, MAPK and other signalling pathways; activates the PI3K/AKT pathway to promote the proliferation and migration of keratinocytes and fibroblasts, and maintains the physiological ratio of collagen type I/III; and upregulates angiogenic factors, including VEGF and bFGF, improving local blood circulation to enhance angiogenesis. Meanwhile, it regulates collagen metabolism, blocks the TGF‐β1/Smad2/3 scar‐related pathway, and counteracts oxidative stress, thereby accelerating wound healing while inhibiting scar formation. In recent years, PNS has shown promising potential in acute trauma, diabetic foot ulcers, burns and scar repair. PLGA/chitosan nanocarriers, hydrogels, electrospun fibrous membranes and smart responsive delivery systems have effectively addressed limitations of traditional dosage forms, including low bioavailability, poor skin permeability and uneven drug release. Nevertheless, research gaps remains: unclear targets of core monomers r; ambiguous pathway interactions among monomers; and poorly understood crosstalk between signalling pathways; studies on the impacts of extraction processes on pharmacodynamic effects are lacking; and high‐level clinical evidence is inadequate. Thus, PNS possesses substantial potential for treating refractory wounds because of its multi‐component synergy and full‐cycle regulation. Future research should focus on in‐depth exploration of molecular mechanisms, standardization of extraction processes, optimization of smart delivery systems and implementation of high‐quality clinical studies to promote the translation of active ingredients from traditional Chinese medicine into modern precision therapies.

## 1 Introduction

The skin is the largest organ in the human body and performs multiple functions, including serving as a physical barrier and providing immune defence. Accordingly, its structural integrity is essential for maintaining overall health [1]. Skin trauma resulting from mechanical, thermal and other causes is classified into acute trauma, chronic wounds and burns, among other categories. Skin wound repair follows four sequential phases—haemostasis, inflammation, proliferation and remodelling—and requires coordinated interactions among diverse cell types and molecular mediators (Figure 1) [2]. While superficial wounds can heal spontaneously, deep or complex wounds are prone to delayed healing, leading to substantial clinical challenges [3]. The global incidence of refractory wounds is estimated to range from 1% to 2%, and this prevalence is increasing in parallel with population ageing and the growing burden of diabetes. Notably, the annual recurrence rate of diabetic foot ulcers exceeds 30%, posing a significant health threat to affected patients [4, 5]. Traditional treatments, including debridement, anti‐infection measures and conventional dressings, are often associated with prolonged healing cycles and an elevated risk of complications, including infection and scar hyperplasia. In addition, topical antibiotics can promote drug resistance, recombinant growth factors require frequent administration and are costly, and surgical skin grafting carries inherent risks. Thus, there is an urgent clinical need for more effective therapeutic strategies to address these limitations [6, 7].

**FIGURE 1:**
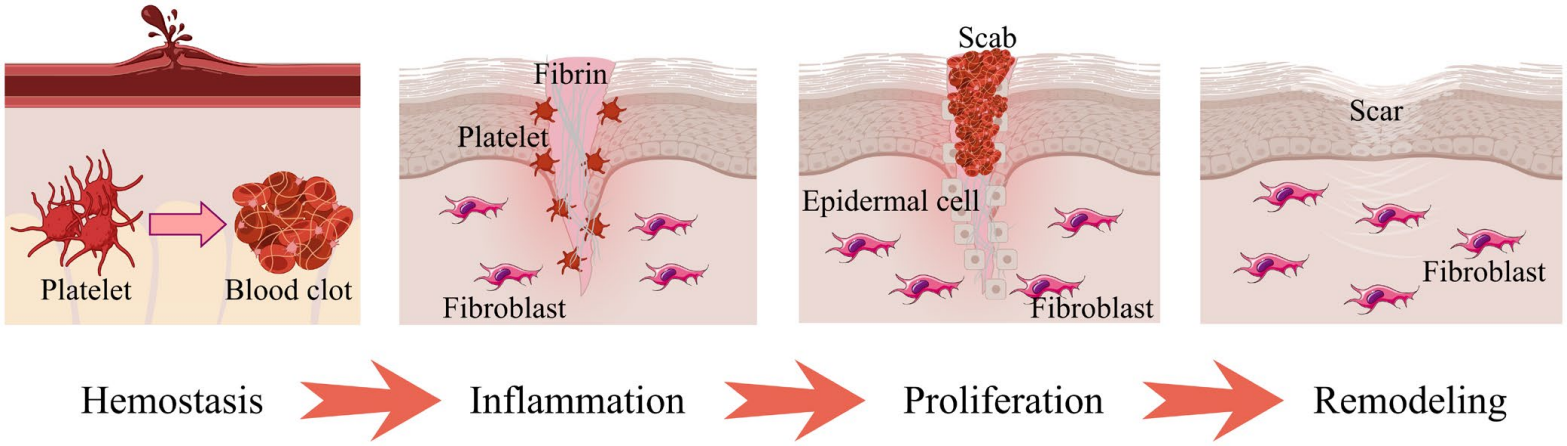
Process of skin wound repair. Skin wound repair is a highly coordinated, dynamic process involving interactions among multiple components, including cells, cytokines and the extracellular matrix (ECM). It is classically divided into four continuous and partially overlapping phases: Haemostasis, inflammation, proliferation and remodelling.

*Panax notoginseng* is a highly valued herb in traditional Chinese medicine, with saponins as its primary active constituents. PNSs comprise multiple monomers, including ginsenosides Rg1 and Rb1, and modern processing technologies allow purification levels exceeding 90% [8]. PNSs exert multitarget modulatory effects across all phases of wound repair, primarily through anti‐inflammatory, antioxidant and pro‐angiogenic activities. Specifically, they modulate the inflammatory microenvironment, accelerate cellular proliferation and migration, and suppress scar formation [9, 10]. In this review, we summarize relevant findings, focusing on the molecular mechanisms by which PNSs modulate skin wound repair. We also evaluate application differences across wound types, innovations in dosage forms and evidence for clinical translation. Our objective is to provide a reference for basic research, dosage form optimization and standardization of the clinical application of PNSs, thereby promoting their precise translation in wound repair.

## 2 Core Mechanisms Underlying the Modulatory Effects of PNSs in Skin Wound Repair

Skin wound repair is a complex physiological process involving the coordinated actions of multiple cells and molecules, and its orderly progression depends on regulation of inflammatory microenvironment homeostasis, proliferation and differentiation of repair cells, angiogenesis and tissue remodelling [11]. PNS are natural active saponin mixtures extracted from *Panax notoginseng*. After purification using modern techniques, the principal active monomers are ginsenoside Rg1 and ginsenoside Rb1, accompanied by minor active components such as ginsenoside Rd., ginsenoside Re, and notoginsenoside R1. Each monomer exerts independent pharmacological activity and, through synergistic interactions, contributes to the overall multitarget and full‐cycle regulatory effects of PNS on wound repair [8]. Most existing studies use total PNS as the research object, and analyses of the specific effects of individual monomers, interactions among monomers, and efficacy differences compared with total PNS remain limited [12]. Building on a systematic discussion of the core mechanisms by which PNS regulates wound repair, this section further clarifies the independent roles of major monomers, their efficacy differences from total PNS, and synergistic and antagonistic relationships among monomers. The regulatory mechanisms of PNS and its key monomers are summarized according to the sequential stages of wound repair, followed by an analysis of consensus conclusions, conflicting results and critical gaps in the current literature.

### 2.1 Modulation of the Inflammatory Phase: Anti‐Inflammatory and Immunomodulatory Effects

The inflammatory response is the initial stage of wound repair. Moderate inflammation facilitates removal of foreign bodies and necrotic tissue from the wound, whereas excessive or persistent inflammation disrupts the reparative microenvironment and delays healing [13]. Existing studies have demonstrated that total PNS can regulate the inflammatory phase through three main mechanisms: inhibiting pro‐inflammatory responses, modulating key signalling pathways and balancing the immune microenvironment, thereby facilitating a smooth transition from the inflammatory phase to the proliferative phase [14, 15]. Ginsenoside Rg1 and ginsenoside Rb1 are the core active monomers underlying the anti‐inflammatory effects of total PNS. These monomers differ substantially in their targets, regulatory signalling pathways and efficacy, and their effects also differ from the overall effects of total PNS.

Regarding inhibition of pro‐inflammatory factors, both in vitro and animal model studies have confirmed that total PNS significantly downregulates mRNA transcription and protein expression of pro‐inflammatory factors, including IL‐6, IL‐8 and TNF‐α, in wound tissues and inflammatory cells, reduces infiltration of inflammatory cells such as neutrophils and macrophages in the local wound area, and prevents excessive inflammation from damaging surrounding normal tissues [16, 17]. Among these, ginsenoside Rg1 is the core monomer responsible for downregulating pro‐inflammatory factors within total PNS. It primarily acts by targeting and inhibiting the NF‐κB pathway and can dose‐dependently reduce transcription and secretion of TNF‐α and IL‐1β [18]. Ginsenoside Rb1 shows more specific inhibitory effects on IL‐6 and IL‐8. It also markedly suppresses mast cell degranulation and reduces release of inflammatory mediators such as histamine and tryptase, compensating for the limited regulation of non‐macrophage‐derived inflammatory mediators by Rg1 [19]. Compared with total PNS, Rg1 or Rb1 alone targets only a subset of pro‐inflammatory factors and inflammatory cells, whereas total PNS blocks the broader inflammatory cascade through multi‐monomer synergy. At equivalent dosages, the overall anti‐inflammatory effect of total PNS is significantly greater than that of individual monomers, with no apparent dose‐dependent toxicity [16, 17]. However, consistent dose–response conclusions have not been established. Low concentrations of total PNS have weak inhibitory effects on macrophage‐derived pro‐inflammatory factors and may even modestly upregulate TNF‐α expression to initiate early inflammatory defence. In contrast, high concentrations of Rg1 alone may inhibit macrophage phagocytosis, which is detrimental to clearance of foreign bodies and pathogens from the wound. This highlights a key advantage of total PNS in achieving balanced effects via the combinatorial ratio of multiple monomers [17, 20].

Regulation of signalling pathways constitutes the core molecular basis for the anti‐inflammatory effects of PNS. Total PNS can simultaneously target two central inflammatory pathways: NF‐κB and MAPK. On the one hand, it blocks NF‐κB pathway activation by inhibiting degradation of IκBα and preventing nuclear translocation of the NF‐κB p65 subunit. On the other hand, it suppresses phosphorylation and activation of ERK1/2, p38 and JNK, thereby interrupting the inflammatory cascade of the MAPK pathway [18, 19, 21, 22, 23]. In contrast, ginsenoside Rg1 exerts highly specific inhibition of the NF‐κB pathway, whereas its effects on the MAPK pathway are weaker. Ginsenoside Rb1 mainly targets the MAPK pathway and mediates bidirectional regulation of p38 and JNK. It inhibits excessive phosphorylation of JNK while moderately activating the ERK1/2 pathway to promote repair, thereby avoiding potential risks of overly rapid inflammation resolution and incomplete clearance of wound foreign bodies that may result from total PNS inhibiting the entire MAPK pathway [19]. However, most studies have only assessed the effects of total PNS on pathway phosphorylation, without identifying direct targets of each monomer or clarifying the synergistic regulatory mechanisms of Rg1 and Rb1 on these pathways within total PNS. Moreover, some pulmonary fibrosis models suggest that total PNS exerts opposite regulatory effects on p38 and JNK within the MAPK pathway, potentially due to differential regulation by Rg1 and Rb1, but this has not been systematically verified in skin wound models [19].

Furthermore, total PNS can upregulate the expression of the anti‐inflammatory factor IL‐10 and induce macrophage polarization from the pro‐inflammatory M1 phenotype to the reparative M2 phenotype, thereby coordinating inflammation suppression with repair initiation [20, 24, 25, 26]. Among these, ginsenoside Rb1 is the key monomer that induces macrophage polarization, and its effect in triggering M2 polarization is more than three times stronger than that of Rg1. It markedly upregulates the expression of M2‐specific markers CD206 and Arg‐1 and simultaneously promote M2 macrophages to secrete reparative factors, including TGF‐β1 and VEGF. In contrast, ginsenoside Rg1 primarily reduces the proportion of pro‐inflammatory macrophages by inhibiting transcription of genes associated with M1 polarization, producing a synergistic ‘anti‐proinflammation and pro‐repair’ effect with Rb1 [20, 24]. Nevertheless, important conflicts and gaps remain. The regulatory effects of PNS on macrophage polarization differ across disease models. Kim et al. [20] reported that total PNS promoted macrophage polarization towards the M1 phenotype in tumour models, whereas studies in wound repair have demonstrated its ability to induce M2 polarization. This difference likely reflects distinct dominant effects of Rg1 and Rb1 across microenvironments: the M1‐polarizing effect of Rg1 predominates in the tumour microenvironment, whereas the M2‐polarizing effect of Rb1 dominates in the wound microenvironment. The overall effect of total PNS depends on the ratio of these two monomers and the local microenvironment, but this mechanism has not been clarified in wound repair research. Meanwhile, current studies primarily focus on macrophages and do not examine effects of other monomers on immune cell subsets such as T lymphocytes and neutrophils, limiting delineation of the overall regulatory network of total PNS in the wound immune microenvironment.

### 2.2 Modulation of the Proliferative Phase: Promotion of Cell Proliferation and Migration

The proliferative phase constitutes the core stage of wound healing, characterized by epithelialization via keratinocyte proliferation and migration and granulation tissue formation through fibroblast activation and extracellular matrix (ECM) synthesis. Accumulating studies have confirmed that total PNS accelerates the proliferative process by regulating the functions of these key reparative cells, with consistent findings across cellular and animal wound models [27, 28, 29, 30, 31]. Ginsenoside Rg1 and ginsenoside Rb1 are the two pivotal monomers underlying the pro‐proliferative effects of total PNS, targeting keratinocytes and fibroblasts, respectively. They differ substantially in their independent roles and cellular tropism and complement the overall effects of total PNS.

With regard to keratinocytes, total PNS is generally considered to act mainly by activating the PI3K/AKT‐ERK1/2 signalling pathway. It promotes the transition of HaCaT cells from the G0/G1 phase to the S phase, increases the percentage of PCNA‐positive cells, and enhances proliferative activity. It also upregulates MMP‐2/9 activity to facilitate ECM degradation, removing spatial barriers to keratinocyte migration and accelerating epithelialization [27, 28]. Ginsenoside Rg1 is the key active component of total PNS in regulating keratinocyte function. It can functionally targets the PI3K/AKT‐ERK1/2 signalling axis, sustain activation of the PI3K/AKT‐ERK1/2 pathway, and significantly shorten the epithelialization period [27]. Notably, the original evidence supporting this regulatory effect was derived from an oral submucous fibrosis model rather than a skin wound‐specific system, and was verified by downstream phosphorylation assays rather than biophysical validation of direct protein‐ligand interaction. In contrast, ginsenoside Rb1 shows extremely weak regulatory effects on keratinocyte proliferation; it only slightly upregulates MMP‐9 activity and exerts no significant influence on the cell cycle. Compared with total PNS, Rg1 alone displays a more pronounced pro‐epithelialization effect and may specifically resolve epithelialization defects in delayed wound healing. Total PNS, however, supports both epithelialization and granulation tissue formation, better matching overall reparative demands during the proliferative phase [28]. Nevertheless, two major limitations remain. First, the physiological relevance of cellular models is limited: most studies use the HaCaT cell line, few use human primary keratinocytes, and none simulate dysfunction under pathological conditions such as diabetes and ageing. Consequently, differential effects of Rg1 and total PNS have not been validated in pathological models. Second, conclusions regarding MMP regulation are conflicting. Total PNS and Rg1 upregulate MMP‐2/9 to promote migration during wound repair, whereas Rb1 significantly downregulates excessive MMP activation to inhibit tissue fibrosis in models such as oral mucosal fibrosis. The conditions and concentration thresholds for this bidirectional regulation remain unclear, and it has not been explained how total PNS balances MMP activity through monomer combinations to achieve both pro‐migratory and anti‐fibrotic effects [27, 29].

As the central cell type in granulation tissue formation, fibroblast activity directly determines wound healing quality. Existing studies suggest that total PNS significantly increases fibroblast proliferation by regulating expression of TGF‐β1 receptors, promotes synthesis and secretion of type I and type III collagen, and maintains their physiological ratio of 3:1, providing stable ECM support and accelerating granulation tissue formation [29, 30, 31]. Ginsenoside Rb1 is the key monomer in total PNS that modulates fibroblast function; it can functionally modulates the activity of the TGF‐β1 type II receptor and exerts bidirectional regulation on fibroblasts. It should be noted that this conclusion was initially extrapolated from a pulmonary fibrosis cell model, and direct receptor‐ligand binding has not been confirmed by biophysical assays in skin wound contexts. In the early proliferative phase, it moderately promotes fibroblast proliferation and collagen synthesis to fill wound defects; in the late proliferative phase, it inhibits excessive fibroblast activation to prevent abnormal collagen deposition [29]. By contrast, ginsenoside Rg1 has no apparent pro‐proliferative effect on fibroblasts and primarily supports fibroblast migration by upregulating MMP activity. Compared with total PNS, Rb1 alone regulates the collagen ratio more precisely and maintains the physiological proportion of type I/III collagen. However, in pathological models such as diabetic wounds, total PNS upregulates type III collagen to a significantly greater extent than type I collagen, potentially deviating from the 3:1 physiological level; this may be related to non‐specific regulation by other monomers in total PNS [32]. Research conflicts and gaps persist. Total PNS and Rb1 exert bidirectional effects on fibroblast proliferation: they promote proliferation of normal fibroblasts in wound repair studies but inhibit excessive proliferation of scar fibroblasts in hypertrophic scar research. Current studies describe these phenotypes separately without clarifying the underlying molecular switch mechanism or the synergistic or antagonistic effects of other monomers in total PNS on this bidirectional regulation [10, 31]. Furthermore, existing research has not explored the influence of PNS on fibroblast heterogeneity or clarified selective effects of Rg1 and Rb1 on different fibroblast subsets, limiting identification of the key target cell populations through which total PNS promotes granulation tissue formation.

### 2.3 Mechanisms by Which PNSs Promote Angiogenesis

Angiogenesis is critical for wound repair. New blood vessels supply oxygen, nutrients and cytokines required for repair, and impaired angiogenesis is a major contributor to chronic refractory wounds. Extensive studies have confirmed that total PNS promotes wound angiogenesis and increases wound microvessel density (MVD) through synergistic effects that include upregulating angiogenic factors, activating signalling pathways and improving blood circulation [14, 32, 33, 34, 35, 36, 37]. Ginsenoside Rg1 is the key monomer underlying the pro‐angiogenic effect of total PNS, whereas Rb1 exerts bidirectional regulation of angiogenesis. Their interactions determine the stability and safety of angiogenesis induced by total PNS, which differs fundamentally from the effects of individual monomers.

Total PNS significantly upregulates transcription and secretion of VEGF and bFGF in HUVECs. These factors synergistically mediate endothelial cell proliferation, migration and lumen formation, thereby driving angiogenesis [32]. Ginsenoside Rg1 is the key component of total PNS in upregulating VEGF. It can dose‐dependently enhance VEGF expression and secretion by activating the HIF‐1α/VEGF pathway, promoting endothelial cell proliferation, migration and tube formation [34]. Ginsenoside Rb1 has no obvious upregulating effect on VEGF but can increase bFGF expression to promote basement membrane formation and neovascular maturation. It also antagonizes the overexpression of VEGF caused by high‐concentration Rg1, thereby reducing risks of increased vascular permeability and aberrant vascular proliferation [34]. Compared with total PNS, Rg1 alone exerts stronger pro‐angiogenic effects and can specifically target angiogenic disorders in chronic wounds, yet tends to cause adverse effects such as vascular leakage and abnormal proliferation at high doses. In contrast, total PNS achieves both angiogenesis promotion and stability through synergy between Rg1 and Rb1, enhancing angiogenesis while maintaining structural and functional integrity of new blood vessels and providing better safety than Rg1 alone [35, 36]. However, current research indicators are limited. Most studies focus on VEGF and bFGF regulation by total PNS and Rg1, with little investigation of factors involved in vascular maturation, such as Ang and PDGF. Using MVD alone cannot fully capture the effects of total PNS and its monomers on neovascular function. In addition, the regulatory effects of different monomers can be contradictory: Rg1 upregulates VEGF to promote angiogenesis, whereas Rb1 inhibits angiogenesis in tumour tissue by downregulating VEGF [34]. Synergistic or antagonistic effects between these monomers within total PNS and differences in angiogenic activity across ratios have not been systematically elucidated, which may contribute to inconsistent results among different batches of PNS studies. Importantly, the pro‐angiogenic effect of total PNS is microenvironment‐dependent: it is significant under normoxic conditions but markedly weakened in the severely hypoxic microenvironment of chronic wounds. This phenomenon relates to Rg1 regulation of the HIF‐1α pathway, but it remains unclear whether other monomers in total PNS can mitigate this microenvironment dependence [32].

The core molecular mechanism by which total PNS promotes angiogenesis involves synergistic activation of two key pathways: JAK2‐STAT3 and PI3K/AKT [35, 36]. Ginsenoside Rg1 primarily activates the PI3K/AKT pathway, upregulates the anti‐apoptotic protein Bcl‐2 in endothelial cells, inhibits endothelial cell apoptosis, and promotes lumen formation. Ginsenoside Rb1 primarily regulates the JAK2‐STAT3 pathway, enhancing endothelial cell migration and adhesion and facilitating the stabilization and maturation of new blood vessels [35, 36]. Compared with monomers, total PNS can activate both pathways to regulate the process of angiogenesis, whereas individual monomers target only specific stages and have less comprehensive effects. However, mechanistic integration remains limited. Substantial crosstalk exists between the JAK2‐STAT3 and PI3K/AKT pathways, yet current studies tend to analyse them separately without clarifying how Rg1 and Rb1 jointly regulate pathway crosstalk or identifying the dominant pathway underlying the pro‐angiogenic effects of total PNS. In addition, studies have not ruled out indirect mechanisms. Total PNS can induce M2 macrophage polarization and indirectly increase secretion of pro‐angiogenic factors such as VEGF [35], but current evidence does not distinguish direct effects of total PNS and its monomers on endothelial cells from indirect effects mediated through the immune microenvironment, limiting precision in mechanistic analysis.

In addition, total PNS improves local blood circulation at the wound site by inhibiting platelet adhesion and aggregation, providing a favourable environment for angiogenesis [32, 34]. Ginsenoside Rg1 is the key monomer underlying the anticoagulant effect of total PNS; it significantly inhibits platelet activation and aggregation and prolongs activated partial thromboplastin time. In contrast, ginsenoside Rb1 has weak anticoagulant activity and primarily improves hemorheological properties by reducing plasma fibrinogen levels [34]. Compared with total PNS, Rg1 alone exerts a stronger anticoagulant effect and carries a higher risk of bleeding when applied topically. Total PNS balances hemostatic and anticoagulant effects through multi‐monomer combinations, making it more suitable for clinical management of acute traumatic wounds and reducing bleeding risk potentially caused by Rg1 alone. Nevertheless, this area has gaps in clinical translation and safety assessment. Current studies have only observed microcirculation‐improving effects of total PNS in animal models and have not verified effects on blood perfusion after topical administration in human wounds. In addition, dose thresholds and optimal timing for anticoagulant effects of topical total PNS and its monomers have not been determined, limiting safety guidance for clinical use.

### 2.4 Modulation of the Remodelling Phase: Tissue Repair and Scar Improvement

The remodelling phase of wound repair centres on ECM remodelling and functional tissue recovery. Imbalanced collagen metabolism or aberrant remodelling at this stage often leads to excessive scar hyperplasia and post‐healing dysfunction. Existing studies have confirmed that total PNS can regulate tissue remodelling by modulating collagen metabolism, inhibiting scar‐related signalling and counteracting oxidative stress, thereby promoting healing while limiting scarring [9, 10, 38, 39, 40]. Ginsenoside Rb1 is the key monomer underlying the anti‐scarring effects of total PNS, whereas Rg1 primarily improves the remodelling microenvironment via anti‐oxidative stress. These two monomers differ significantly in targets and activity and together contribute to the overall regulatory effects of total PNS.

Regulation of collagen metabolic homeostasis by total PNS is mainly achieved through bidirectional modulation of fibroblast function. On the one hand, it inhibits excessive fibroblast proliferation and reduces abnormal collagen deposition; on the other hand, it promotes orderly collagen reorganization and regulates the MMPs/TIMP‐1/2 balance to maintain dynamic equilibrium between ECM synthesis and degradation [27, 38]. Ginsenoside Rb1 is the key monomer in total PNS that regulates collagen metabolism. It bidirectionally controls collagen synthesis and degradation in fibroblasts: promoting orderly collagen synthesis during the proliferative phase and suppressing excessive collagen deposition during the remodelling phase, while precisely tuning the MMPs/TIMP balance to avoid disordered ECM degradation. In contrast, ginsenoside Rg1 exerts no direct regulatory effect on collagen synthesis and primarily facilitates collagen degradation and remodelling by upregulating MMP‐2/9 activity [27, 38]. Compared with total PNS, Rb1 alone regulates collagen metabolism more precisely and exhibits a more prominent anti‐scarring effect. Total PNS, however, covers collagen synthesis, degradation and remodelling, better matching overall repair requirements in the remodelling phase. Nevertheless, contradictions and gaps remain. The bidirectional regulatory mechanism by which total PNS and its monomers influence collagen synthesis has not been fully elucidated: collagen synthesis is promoted during the proliferative phase but excessive collagen deposition is inhibited during the remodelling phase. Current studies have not defined the temporal window, concentration threshold, or molecular switch underlying this effect, nor clarified synergistic mechanisms among monomers in total PNS, limiting the theoretical basis for stage‐specific clinical administration [10, 31]. In addition, findings on MMPs/TIMP regulation by total PNS are inconsistent. Some studies report that total PNS upregulates TIMP‐1/2 to restrain excessive MMP activation [27], whereas others report increased MMP activity to promote collagen remodelling [38]. This discrepancy likely reflects distinct dominant effects of Rg1 and Rb1 at different healing stages, but this mechanism has not been systematically analysed. Notably, most studies measure only type I/III collagen expression, without systematically assessing scar‐related indicators such as collagen cross‐linking, fibre arrangement and mechanical properties or comparing regulatory differences between total PNS and individual monomers on collagen structure, which limits evaluation of their effects on tissue remodelling.

A central mechanism of scar formation is fibroblast‐to‐myofibroblast transition, for which the TGF‐β1/Smad2/3 pathway is a key regulator. Accumulating evidence indicates that total PNS blocks binding of TGF‐β1 to its receptors and inhibits phosphorylation and nuclear translocation of Smad2/3, thereby reducing fibroblast‐to‐myofibroblast transition, downregulating α‐SMA expression and alleviating scar hyperplasia [10, 29]. Ginsenoside Rb1 is the principal active component of total PNS responsible for anti‐scarring effects; it can exerts a competitive inhibitory effect on the TGF‐β1 type II receptor at the functional level and block Smad2/3 pathway activation [10]. Currently, this inhibitory effect is mainly supported by pharmacological functional assays, and direct biophysical evidence of protein–ligand binding in skin wound tissue remains lacking. Ginsenoside Rg1 does not directly inhibit the TGF‐β1/Smad2/3 pathway and instead indirectly reduces α‐SMA expression through anti‐oxidative stress to assist in anti‐scarring. Compared with total PNS, Rb1 alone shows more targeted anti‐scarring effects and can more specifically suppress scar hyperplasia, whereas total PNS acts more mildly, promoting healing while inhibiting scarring and reducing the risk of decreased wound healing strength potentially associated with high‐concentration Rb1 [9]. However, two major limitations remain. First, mechanistic analysis lacks precision: existing studies describe inhibitory effects of total PNS and Rb1 on the TGF‐β1/Smad2/3 pathway but do not identify direct targets or clarify synergistic or antagonistic of other monomers in total PNS. Second, the clinical relevance of models is limited: most studies adopt in vitro scar fibroblasts and rodent scar models, yet wound healing in rodents relies largely on contraction and differs substantially from human scarring mechanisms. Differential effects of total PNS and its monomers in human scar tissue have not been validated, and large‐sample clinical trials are lacking.

Oxidative stress is a major contributor to abnormal tissue remodelling. After trauma, excessive accumulation of reactive oxygen species (ROS) at the wound site impairs reparative cell function and exacerbates collagen metabolic disorders. Existing studies indicate that total PNS has substantial antioxidant capacity. It enhances ROS scavenging by increasing activities of SOD and GSH‐Px, reduces MDA accumulation and alleviates oxidative cellular injury [32, 39, 40]. Ginsenoside Rg1 is the key monomer responsible for the anti‐oxidative effect of total PNS and can protect fibroblast and endothelial cell function during remodelling. By contrast, ginsenoside Rb1 has weak antioxidant activity and only slightly increases GSH‐Px activity [39, 40]. Compared with total PNS, Rg1 alone exerts stronger antioxidant effects and may specifically improve the oxidative microenvironment in pathological wounds such as diabetic and aged wounds. Total PNS can simultaneously provide antioxidant and anti‐scarring effects, making it more suitable for overall repair demands during remodelling. However, the evidence remains largely phenomenological. The core molecular mechanisms underlying antioxidant effects of total PNS and Rg1 remain unclear. Most studies only measure oxidative stress markers and do not determine whether effects reflect direct ROS scavenging or activation of endogenous antioxidant pathways such as Nrf2/HO‐1. In addition, the contribution of antioxidant effects to remodelling regulated by total PNS and its monomers has not been established, making it difficult to determine whether anti‐scarring effects derive from direct inhibition of the TGF‐β1 pathway or indirect protection through antioxidant activity. Moreover, antioxidant and remodelling effects of total PNS and its monomers have not been systematically compared in pathological wound models with markedly elevated oxidative stress, such as diabetic and aged wounds, and their anti‐scarring value in chronic refractory wounds remains insufficiently explored.

### 2.5 Systematic Analysis of Effect Comparison and Synergistic/Antagonistic Interactions Between Core Monomers of *Panax notoginseng* Saponins and Total Saponins

As the two most abundant and biologically active core monomers in PNS, ginsenoside Rg1 and ginsenoside Rb1 exert distinct independent effects and show different targeting specificities throughout the entire cycle of wound repair. Through synergistic interactions, they form the overall multi‐target and full‐cycle regulatory effect of total PNS, while also presenting remarkable differences from total PNS in clinical application value.

#### 2.5.1 Synergistic Effects Between Core Monomers

The overall effect of total PNS in wound repair is not a simple superposition of the pharmacological activities of individual monomers, but an enhancement of efficacy achieved through multidimensional synergy between Rg1 and Rb1. Rg1 and Rb1 target core signalling pathways at different wound repair stages: Rg1 mainly contributes to early inflammation inhibition, accelerated epithelialization and initiation of angiogenesis, whereas Rb1 supports the transition from inflammation to proliferation, granulation tissue formation, vascular maturation and scar suppression. Their pathway targeting is complementary, covering key nodes across all four wound repair stages and forming the multitarget, full‐cycle regulatory network of total PNS, thereby addressing the limitation that single monomers affect only parts of the repair process. Rg1 and Rb1 also balance each other's effect magnitude and potential risks, supporting stability and safety of total PNS. Rb1 antagonizes excessive VEGF expression and vascular leakage risk induced by high‐concentration Rg1 and balances the over‐inhibition of inflammation by Rg1 to avoid impairing early foreign body clearance at the wound site. Rg1 compensates for the insufficient pro‐epithelialization effect of Rb1 and reduces the excessive inhibition of fibroblasts by high‐concentration Rb1 to prevent decreased wound healing strength. This balanced synergy enables total PNS to exert stable therapeutic effects across a wide dosage range and significantly reduces the risk of adverse reactions, representing a central clinical advantage of total PNS compared with single monomers. Combined administration of Rg1 and Rb1 can produce synergistic enhancement. In vitro studies show that when Rg1 and Rb1 are administered in the natural ratio present in PNS, overall effects on anti‐inflammation, endothelial tube formation, and anti‐scarring are significantly higher than the sum of effects of Rg1 or Rb1 alone at equivalent doses. The two monomers can enhance each other's pharmacological activities by upregulating expression of each other's target receptors, thereby achieving synergistic efficacy [8, 10].

#### 2.5.2 Potential Antagonistic Effects Between Monomers and Research Gaps

Existing studies also suggest potential antagonistic effects among PNS monomers under specific conditions, which may contribute to inconsistent findings across studies. Under extreme pathological conditions, such as the tumour microenvironment and hyperinflammatory responses, the dominant effects of Rg1 and Rb1 may become antagonistic. In tumour models, Rg1 promotes M1 macrophage polarization and exerts antitumor effects, whereas Rb1 induces M2 macrophage polarization, producing opposing effects [20]. At high doses, Rb1‐mediated downregulation of VEGF may antagonize the pro‐angiogenic effects of Rg1, resulting in weaker pro‐angiogenic activity of total PNS than Rg1 alone [34]. However, whether such antagonism occurs under conventional doses and within physiological or pathological microenvironments of skin wound repair has not been systematically verified. Both Rg1 and Rb1 are metabolized by the hepatic CYP450 enzyme system and share metabolic isoenzymes. At high concentrations, metabolic competition may alter their respective in vivo half‐lives and bioavailability, affecting overall efficacy of total PNS [8]. Nevertheless, current studies are largely limited to in vitro pharmacodynamic investigations, without analysing their in vivo metabolic interactions or clarifying their effects on bioavailability after topical wound administration, representing a major gap in current research.

#### 2.5.3 Differences in Clinical Application Value Between Total PNS and Single Monomers

As a multi‐component mixture with a natural constituent ratio, total PNS exhibits comprehensive, stable efficacy and high safety, making it suitable for full‐cycle conventional treatment of various acute and chronic skin wounds without frequent adjustment of administration regimens according to different healing stages. This supports convenient clinical operation and high acceptance among medical staff and patients, making total PNS a practical option for routine wound repair therapy. In contrast, single monomers have higher target specificity and can achieve precise interventions aimed at key pathological processes across wound types and healing stages: for wounds with delayed epithelialization, Rg1 may be used to accelerate epithelialization; for wounds at high risk of scarring, Rb1 may be used during remodelling to suppress scar hyperplasia; and for chronic wounds such as diabetic foot ulcers, staged administration of Rg1 and Rb1 may be implemented based on wound healing conditions to realize individualized precise therapy (Table 1).

**TABLE 1:** Systematic comparison of effects of *Panax notoginseng* saponins and its core monomers in skin wound repair.

Regulatory dimension	Overall effect of *Panax notoginseng* saponins (PNS)	Independent effect of ginsenoside Rg1	Independent effect of ginsenoside Rb1	Correlation between the two monomers and total PNS	References
Core targeted pathways	Synergistic regulation of multiple pathways including NF‐κB, MAPK, PI3K/AKT, JAK2‐STAT3 and TGF‐β1/Smad2/3	Specifically targets NF‐κB and PI3K/AKT pathways, mainly regulating inflammation inhibition, epithelialization, angiogenesis and anti‐oxidative stress	Specifically targets MAPK, TGF‐β1/Smad2/3 and JAK2‐STAT3 pathways, mainly regulating macrophage polarization, collagen metabolism, anti‐scarring and vascular maturation	The multipathway regulatory effect of total PNS entirely relies on the complementary pathway targeting of Rg1 and Rb1, which account for more than 90% of the pharmacological activity of total PNS	[8, 10, 18, 19, 20, 21, 22, 23, 29, 33, 34]
Key effects in the inflammatory stage	Comprehensively inhibits the pro‐inflammatory cascade, induces M2 macrophage polarization, balances inflammation initiation and resolution, with stable and holistic effects	Mainly inhibits the NF‐κB pathway, downregulates pro‐inflammatory factors such as TNF‐α and IL‐1β, suppresses M1 macrophage polarization, with rapid and potent anti‐inflammatory effects	Mainly inhibits the MAPK pathway, downregulates pro‐inflammatory factors such as IL‐6 and IL‐8, strongly induces M2 macrophage polarization, and promotes the transition from inflammation to the proliferative stage	Synergistically achieve the dual effects of ‘suppressing pro‐inflammation and promoting repair’ of total PNS. Rg1 compensates for the insufficient early anti‐inflammatory effect of Rb1, while Rb1 balances the excessive inflammation inhibition by Rg1 to avoid impairing the clearance of foreign bodies in the wound	[13, 14, 15, 16, 17, 18, 19, 20, 21, 22]
Key effects in the proliferative stage	Simultaneously promotes keratinocyte proliferation and migration as well as fibroblast activation, accelerates epithelialization and granulation tissue formation synchronously, and comprehensively advances the proliferative process	Potently promotes keratinocyte proliferation and migration and accelerates epithelialization; serves as the key component of total PNS for promoting epithelialization, with no obvious pro‐proliferative effect on fibroblasts	Precisely regulates fibroblast function, promotes collagen synthesis and maintains the physiological ratio of collagen type I/III, facilitates granulation tissue formation, with no significant effect on keratinocytes	The two monomers synergistically enable total PNS to synchronously promote epithelialization and granulation tissue formation. Rg1 and Rb1 respectively target the two key reparative cell types in the proliferative stage with complementary effects, constituting the core basis for the regulatory role of total PNS in the proliferative phase	[27, 28, 29, 30, 31, 32]
Key effects on angiogenesis	Simultaneously promotes the proliferation, migration and tube formation of vascular endothelial cells as well as neovascular maturation, with stable pro‐angiogenic effects and no risk of excessive proliferation	Markedly upregulates VEGF expression, promotes endothelial cell proliferation, migration and tube formation, showing strong pro‐angiogenic activity; serves as the core component of total PNS for promoting angiogenesis	Upregulates bFGF expression, promotes the maturation and stability of new blood vessels and antagonizes excessive VEGF expression caused by Rg1 at high concentrations to prevent abnormal vascular proliferation	The two monomers synergistically form the vascular regulatory effect of total PNS characterized by ‘promoting angiogenesis + maintaining stability’. Rb1 significantly reduces the risk of vascular leakage induced by high‐concentration Rg1, and the safety of total PNS is remarkably superior to that of Rg1 monomer alone	[14, 32, 33, 34]
Key effects in the remodelling stage	Simultaneously achieves balanced regulation of collagen metabolism, scar inhibition and anti‐oxidative stress, with the dual advantages of promoting healing and resisting scarring, exhibiting mild and comprehensive effects	Potently exerts anti‐oxidative stress, elevates the activities of SOD and GSH‐Px in wound tissues, scavenges ROS, improves the microenvironment during remodelling and assists in promoting orderly collagen remodelling	Potently blocks the TGF‐β1/Smad2/3 pathway, inhibits myofibroblast transformation, reduces excessive collagen deposition and shows significant anti‐scarring effects; serves as the core component of total PNS for anti‐scarring	The two monomers synergistically support total PNS in promoting healing and inhibiting scarring. Rg1 improves the oxidative microenvironment in the remodelling stage and provides a foundation for the anti‐scarring effect of Rb1. Total PNS can avoid the risk of reduced wound healing strength caused by high‐dose Rb1 alone	[9, 10, 27, 38, 39, 40]
Clinical suitability	Suitable for holistic treatment throughout the entire wound repair cycle; can be routinely used for full‐course intervention of various acute and chronic wounds, with a wide range of clinical application scenarios	Suitable for precise intervention at specific healing stages; mainly used for chronic wounds with epithelialization disorders, insufficient angiogenesis and severe oxidative stress injury, allowing staged targeted administration	Suitable for precise intervention of high‐scar‐risk wounds; mainly used for the prevention and treatment of hypertrophic scars and chronic wounds with collagen metabolic disorders, showing significant advantages when administered in the remodelling stage	Total PNS is suitable for routine full‐course treatment of wounds, whereas single monomers enable precise administration targeting core problems in different wound types and healing stages. Together they form a complementary system of ‘routine therapy plus precise intervention’	[8, 9, 10, 32, 34, 41, 42]

Collectively, as the key active monomers in PNS, ginsenoside Rg1 and ginsenoside Rb1 show distinct target specificity and complementary effects across the full process of skin wound repair. Through multiple synergies including complementary pathway targeting, balanced efficacy and risk, and enhanced pharmacological activity, they constitute the core basis for PNS to regulate wound repair in a multitarget and full‐cycle manner. This also explains why total PNS provides more comprehensive, stable and safer therapeutic advantages than individual monomers. Although existing studies suggest potential antagonistic effects between these monomers under extreme pathological conditions and high‐dose administration, key questions—including their action patterns in physiological and pathological microenvironment of wounds and their in vivo metabolic interactions—remain unresolved and represent major gaps in this field. The differentiated clinical application framework of ‘full‐cycle routine treatment plus staged precise intervention’, combining total PNS with single monomers, provides a new strategy for individualized treatment of acute and chronic skin wounds. Further studies are needed to identify direct action targets of monomers, define molecular mechanisms of synergistic and antagonistic effects, and compare clinical efficacy and safety to support translation of PNS from conventional total saponin use to precise, individualized monomer‐targeted therapy (Figure 2).

**FIGURE 2:**
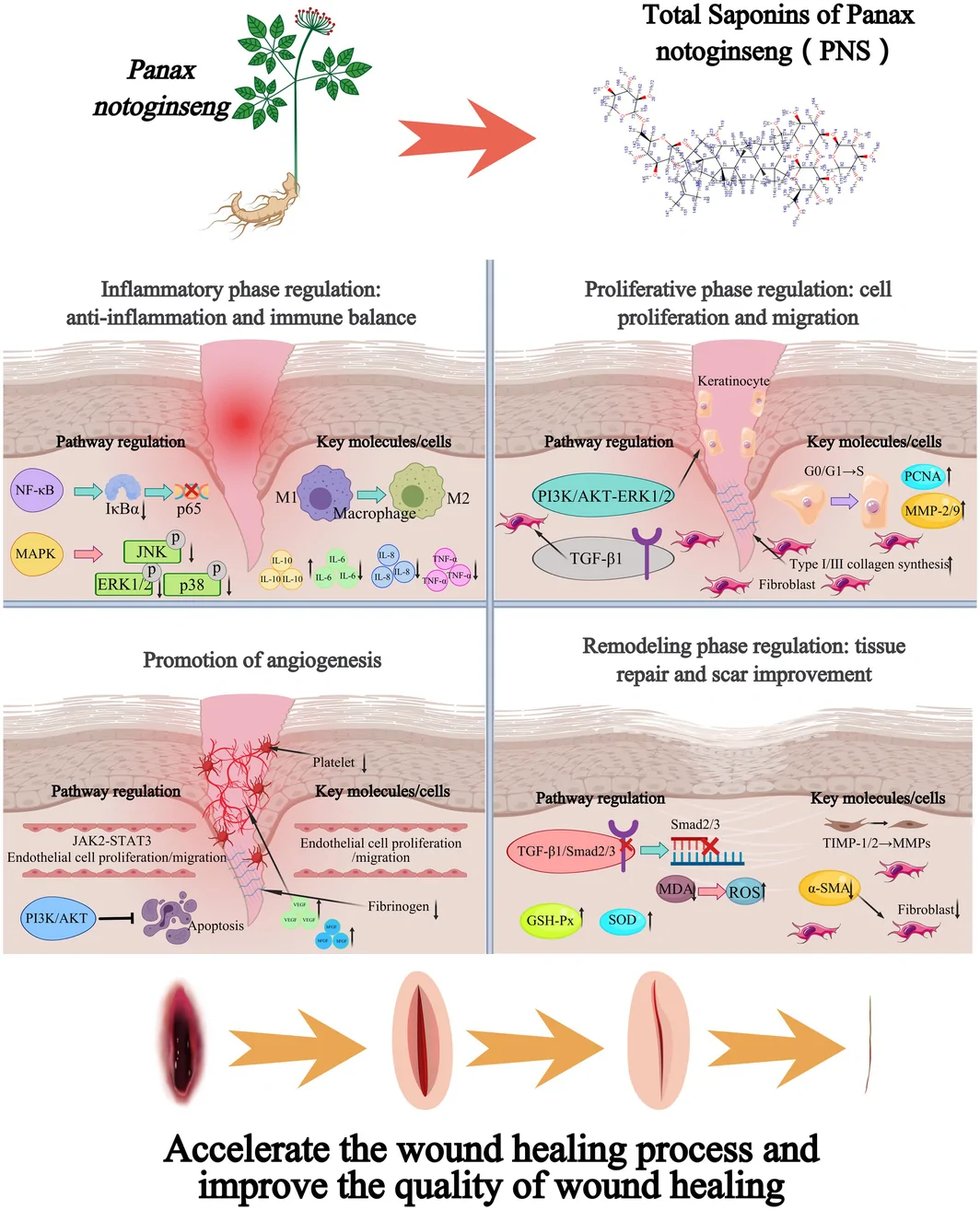
Schematic diagram of multistage molecular mechanisms by which *Panax notoginseng* saponins (PNSs) modulate skin wound repair. PNSs function as core regulatory agents, acting synergistically on wound repair via multitarget and multipathway effects. During the inflammatory phase, PNSs balance the immune microenvironment and induce macrophage polarization. In the proliferative phase, they promote keratinocyte proliferation and migration and support fibroblast‐mediated collagen synthesis, accelerating epithelialization and granulation tissue formation. During angiogenesis, PNSs upregulate angiogenic factors such as vascular endothelial growth factor (VEGF) and basic fibroblast growth factor (bFGF), activate related signalling pathways, increase microvessel density (MVD) and improve local circulation. During remodelling, they maintain collagen metabolic balance, inhibit the transformation of fibroblasts into myofibroblasts, and enhance antioxidant capacity by upregulating the activities of superoxide dismutase (SOD) and glutathione peroxidase (GSH‐Px) while downregulating malondialdehyde (MDA) levels, thereby promoting orderly tissue remodelling and alleviating scar hyperplasia. The synergistic integration of these multidimensional modulatory effects ultimately accelerates the wound healing and improves healing quality.

## 3 Research Progress on Applications of PNSs in Skin Wound Repair

As mechanisms underlying PNS‐mediated wound repair have become clearer, application research has expanded from basic pharmacological exploration to targeted interventions for diverse wound pathologies. In parallel, dosage form innovation and delivery‐system optimization have strengthened the potential for clinical translation of PNSs. Based on recent findings, this section summarizes progress in PNS applications across three dimensions: adaptation to specific wound types, innovations in dosage forms and emerging clinical evidence.

### 3.1 Potential Applications in Different Wound Types

PNSs have demonstrated targeted therapeutic effects in diverse wound conditions, including acute trauma, chronic wounds and burns, attributable to their multistage modulatory properties. Topical administration allows PNSs to act directly at the wound site, thereby improving intervention precision [43].

In acute trauma repair, topical PNS formulations are the primary mode of application and can accelerate wound closure and enhance healing quality. In rat dorsal incision models, PNS cream shortened healing time relative to normal saline and increased tensile strength of healed tissue. This improvement was associated with the ability of PNS to promote collagen synthesis and fibroblast activation [10]. Preclinical studies indicate that PNSs rapidly modulate inflammation, promote re‐epithelialization and reduce infection risk after acute trauma. Accordingly, PNSs may be suitable for wounds requiring rapid healing, such as surgical incisions and traumatic injuries, providing a new option for postoperative wound management [44].

Treatment of chronic wounds is a primary focus of PNS application research, with particularly notable effects reported in diabetic foot ulcers and pressure ulcers. Because of systemic glucose dysregulation, microcirculatory disturbances, and a persistent, imbalanced inflammatory microenvironment, diabetic foot ulcers have low healing rates under conventional treatments. However, PNS composite hydrogels optimize wound conditions through a multifaceted approach, including regulating local inflammation, ameliorating the hypoxic wound microenvironment, and mitigating the inhibitory effects of hyperglycemia on reparative cell function [32, 45]. In preclinical studies, wound healing rate following PNS hydrogel intervention was significantly higher than with normal saline. Furthermore, the structural regularity of granulation tissue and regeneration of skin appendages, such as hair follicles and sebaceous glands, were significantly improved with PNS hydrogel application [46]. In pressure ulcers, PNSs may alleviate ischemia caused by prolonged compression by promoting angiogenesis and granulation tissue expansion. Preclinical data suggest shorter healing cycles with PNS treatment than with control conditions, supporting its potential value for managing pressure ulcers in bedridden patients [47].

Application of PNSs in burn and scar repair focus on comprehensive intervention, including inflammation control, wound healing and scar inhibition. In superficial second‐degree burns, topical PNS formulations have been shown to upregulate the protein and mRNA levels of PDGF‐BB/PDGFR‐β, thereby advancing the peak of growth factor activity and accelerating granulation tissue proliferation and epithelial repair. These effects shorten eschar shedding time and hair regrowth, significantly improving the wound healing rates [48]. In addition, in vitro studies using human hypertrophic scar fibroblasts (HSFs) show that the synthesis of type I/III collagen following PNS treatment is significantly reduced compared with control treatments. Furthermore, the migration of HSFs and the expression of α‐SMA are markedly decreased, effectively inhibiting the formation of hypertrophic scars [10]. In mouse models of post‐traumatic scarring, topical PNS can accelerate wound healing and inhibit scar formation by suppressing excessive fibroblast proliferation and downregulating α‐SMA expression. Furthermore, the scar area in mice treated with PNSs is significantly smaller than in saline‐treated controls, with improved tissue repair quality and no observable local adverse reactions during the experimental period [9].

### 3.2 Dosage Form Innovation and Delivery System Optimization

Limitations of traditional dosage forms are major factors restricting clinical application of PNS. In recent years, breakthroughs in the research and development of novel delivery systems have addressed problems such as low bioavailability and poor targeting, providing a foundation for clinical translation of PNS. After oral administration, traditional PNS preparations can be degraded by gastric acid and intestinal enzymes, resulting in low bioavailability and difficulty achieving effective local concentrations at wound sites [49]. Conventional topical preparations have poor skin penetration and uneven drug release, limiting sustained drug action and compromising therapeutic efficacy [50]. Therefore, developing novel delivery systems with wound targeting, controlled release and long‐acting properties has become a central direction in PNS application research. Most studies focus on pharmacodynamic verification of PNS preparations, with limited comparative analysis of physicochemical properties, biocompatibility and wound adaptability of carrier materials. Building on the core advantages of various delivery systems, this section systematically supplements the advantages, disadvantages and performance differences of different carrier materials, as well as their adaptability to PNS delivery, and analyzes their clinical suitability, large‐scale production potential and core issues related to clinical translation.

#### 3.2.1 Nanocarrier Delivery Systems

Nanocarriers can improve the skin penetration efficiency of PNS through nanoscale effects, enabling targeted drug delivery and long‐acting controlled release, and are among the primary approaches used to address low topical bioavailability of PNS. PLGA nanoparticles and chitosan nanoparticles are the most widely used carriers. They differ substantially in physicochemical properties, drug‐release behaviour and biocompatibility and are suitable for different wound scenarios.

PLGA nanoparticles are prepared using the double‐emulsion solvent evaporation method and have controllable particle size, high encapsulation efficiency and low burst release. In vitro studies show sustained drug release, which improves PNS biocompatibility and stability and reduces adverse reactions from excessively high local concentrations [9]. As a synthetic biodegradable polymer, a key advantage of PLGA is that its degradation rate can be tuned by adjusting the monomer ratio of lactic acid and glycolic acid. A degradation cycle of 1–6 months can be designed to match the wound‐healing cycle, aligning with medication requirements across repair stages. In addition, PLGA has high mechanical strength and chemical stability and is less prone to rupture in the protein hydrolase‐rich humid microenvironment of wounds. It can stably encapsulate lipophilic saponin monomers in PNS and reduce premature drug leakage. PLGA has been approved by the U.S. FDA and China's NMPA for marketed products such as medical implants and injections, with established biosafety and a defined translational pathway. However, PLGA has notable limitations. First, its degradation products (lactic acid and glycolic acid) lower the pH of the local microenvironment and may aggravate local wound inflammation, making it less suitable for infected or highly inflamed wounds. Second, PLGA has no intrinsic biological activity and primarily functions as a drug carrier without additional synergistic effects on wound repair. Third, raw material costs are high, preparation is complex and large‐scale production requires stringent equipment and process control, leading to large variations in encapsulation efficiency between batches [51].

Chitosan nanoparticles, through bioadhesion and penetration‐enhancing effects, can open tight junctions between epithelial cells, promote PNS transport across the skin barrier, and improve local bioavailability, making them particularly suitable for drug delivery in deep wounds [52]. As a naturally derived polysaccharide polymer, chitosan offers several advantages. First, it has intrinsic broad‐spectrum antibacterial and hemostatic activity and can promote mucosal repair. When combined with PNS, it may provide synergistic antibacterial and repair promotion effects, which is relevant for the treatment of infected wounds and acute hemorrhagic trauma. Second, its strong bioadhesion enables tight binding to damaged tissues, prolonging drug retention at the wound site and improving local bioavailability. Third, it is widely available, low cost, and can be prepared under mild conditions without complex equipment, supporting large‐scale production. Fourth, its degradation product is glucosamine, with no risk of local acid accumulation, superior biocompatibility and no aggravation of wound inflammation. However, chitosan nanoparticles also have limitations. First, chitosan has poor water solubility and dissolves mainly under acidic conditions, which can lead to aggregation in neutral physiological environment, resulting in insufficient formulation stability. Second, the burst release effect is substantial, with approximately 30%–50% of the drug released within 24 h after administration, limiting sustained release of PNS. Third, it has low mechanical strength and is prone to rapid swelling and rupture in a high‐wound‐exudate environment, increasing the risk of drug leakage and poor adaptability to deep or highly exudative wounds [52, 53].

From the perspective of clinical feasibility, both types of carriers have established foundations in medical applications. PLGA nanoparticles are more suitable for scenarios requiring long‐term, stable drug release, such as chronic refractory wounds and scar repair, whereas chitosan nanoparticles are better suited to scenarios requiring rapid onset and synergistic antibacterial activity, such as acute hemorrhagic trauma and superficial infected wounds. However, existing research remains insufficient. Pharmacokinetic studies of PNS nanoformulations in damaged human skin are lacking, making it impossible to determine the drug absorption through wounds, in vivo metabolic pathways, and long‐term accumulation risks. Regarding large‐scale production, both carriers have potential for industrial scale‐up. PLGA formulations must address encapsulation‐efficiency uniformity during process scale‐up, whereas chitosan systems require material modification to improve storage stability and controlled‐release performance [51, 53].

#### 3.2.2 Hydrogel Preparations

Hydrogels are hydrophilic polymer materials with three‐dimensional crosslinked network structure, which can swell in water without dissolving, absorb large amounts of wound exudate and maintain a moist wound healing environment. Owing to their high compatibility with the skin wound microenvironment, they have become a preferred dosage form for topical administration of PNS. Hydrogel matrices used for PNS delivery are generally divided into natural polymer hydrogels and synthetic polymer hydrogels. Chitosan‐based hydrogels and thermosensitive PNIPAM hydrogels are the most extensively studied and differ in mechanical properties, swelling behaviour, responsive characteristics and wound adaptability.

Chitosan/PNS composite hydrogels are mostly prepared using freeze‐drying and feature high porosity with moisturizing and controlled‐release functions. Their three‐dimensional porous structure can simulate the ECM and provide a scaffold for fibroblast and keratinocyte adhesion and proliferation [54]. As a naturally derived matrix, chitosan‐based hydrogels offer several advantages, including high biocompatibility and complete biodegradability with non‐cytotoxic degradation products that do not trigger wound foreign body reactions. They also possesses multiple biological functions including moisturizing, exudate absorption, antibacterial activity and procoagulant effects, which synergize with the repair‐promoting effects of PNS. They are the only hydrogel matrix described that can simultaneously provide dressing barrier, drug delivery and synergistic repair functions. In addition, they can be modified via chemical crosslinking and physical blending to adjust porosity, swelling ratio and degradation rate, supporting use in wounds of different depths. With low raw material costs and mature preparation technology, chitosan‐based matrices are widely used in marketed wound dressings, with a clear clinical translation pathway. However, they have important limitations: poor mechanical properties, high brittleness and low tensile strength reduce suitability for wounds at highly mobile sites such as joints, and they are prone to damage and shedding during use. After forming at room temperature, their shape cannot be readily changed, limiting fit to irregular wounds. In addition, rapid swelling may cause overexpansion in highly exudative wounds, leading to rapid drug release and insufficient controlled‐release performance [54, 55].

Thermosensitive PNS hydrogels typically use PNIPAM as the core thermosensitive matrix and are prepared by free‐radical polymerization. They remain liquid at room temperature and rapidly undergo in situ gelation at body temperature after application, facilitating administration, improving fit to the wound surface and reducing dressing‐change frequency [56]. Key advantages of these thermosensitive synthetic polymer hydrogels include excellent in situ gelation properties, can fully fill all dead spaces of lacunar and irregular wounds when administered in liquid form, and form seamless adhesion with wounds after gelation, reducing secondary injury during dressing changes and improving patient compliance. Their mechanical properties can be regulated by adjusting monomer ratios to produce stretchable, resilient hydrogels suitable for highly mobile sites such as joints and the face. Meanwhile, their thermosensitive response characteristics are precise, and the phase transition temperature can be controlled at 32°C–35°C by adjusting the monomer composition, achieving precise regulation of liquid state at room temperature and rapid gelation at body temperature. They also provide controlled release: release rates can be regulated by adjusting the crosslinking density, enabling stable drug release for 1–2 weeks without obvious burst release. Nevertheless, such hydrogels also have limitations. The synthetic matrices have no inherent biological activity and mainly serve as drug carriers without additional synergistic effects on wound repair. Residues of some synthetic monomers may have potential cytotoxicity, requiring stringent purification for medical‐grade materials. In addition, degradation rates may not match wound‐healing cycles, and excessively slow degradation cycle of some synthetic hydrogels may hinder wound epithelialization. In addition, high raw material costs, stringent cross‐linking requirements during preparation, and challenges in quality control during large‐scale production further limit application [56, 57].

Regarding clinical suitability, chitosan‐based hydrogels are more suitable for full‐cycle treatment of conventional wounds such as acute trauma, superficial burns and pressure ulcers and may be suitable as routine dressings. Thermosensitive hydrogels are better suited to scenarios that are difficult to cover with traditional dressings, such as lacunar wounds, sinuses and irregular wounds at joint sites. Preclinical studies suggest that both types of hydrogels loaded with PNS can significantly improve wound healing quality in rat diabetic foot ulcer models [46]. For large‐scale production, industrial technologies for both preparations are established. Chitosan‐based hydrogels should prioritize improving mechanical properties through crosslinking modification, whereas thermosensitive hydrogels should address raw material purity and compatibility with sterilization processes to avoid damage to the three‐dimensional structure of hydrogels and thermosensitive active ingredients of PNS caused by moist heat or irradiation sterilization [55].

#### 3.2.3 Electrospun Fibrous Membranes

Electrospun fibrous membranes are ultrafine fibrous network materials prepared by high‐voltage electrospinning, with fibre diameters ranging from nanometres to micrometres. Their structure closely resembles the natural extracellular matrix of skin, making them a major focus in wound dressings and drug delivery. Polycaprolactone (PCL) is the primary electrospinning matrix for PNS delivery and can be blend‐spun with natural materials such as chitosan and gelatin. These matrices differ in physicochemical properties, biocompatibility and degradation behaviour.

PCL/PNS electrospun fibrous membranes are the main dosage form in this area. With controllable fibre diameters, the fibrous network can guide ordered growth of reparative cells. These membranes enable sustained PNS release and can be co‐loaded with silver ions, antimicrobial peptides and other components to create synergistic repair and anti‐infection systems, which may be suitable for wounds at high risk of infection [58]. As an electrospinning matrix, PCL has several advantages, including excellent spinnability that supports uniform, bead‐free ultrafine fibres across broad process parameters and tunable fibre diameter and porosity (often exceeding 90%). This ensures good gas and water vapour permeability while effectively blocking external bacteria and dust, perfectly matching the barrier requirements of wound dressings. Meanwhile, it has outstanding mechanical properties, high flexibility and tensile strength, can withstand repeated stretching and friction at joint sites without easy breakage. PCL can also be cut into different shapes, supporting clinical use. In addition, PCL degrades slowly, with an in vivo degradation cycle of approximately 2–4 years, enabling long‐term release with limited burst release, which may be useful for chronic refractory wounds. PCL has been approved by the FDA and NMPA for products such as sutures and implantable dressings, and multiple PCL‐based electrospun dressings have been approved for marketing, with a fully established translation pathway [58]. However, PCL also has limitations. It is hydrophobic and has limited cell adhesion, and its ability to recruit and promote the proliferation of reparative cells is weak when electrospun alone, so blending with hydrophilic natural materials such as chitosan and gelatin is often required. Its slow degradation cycle means fibrous membranes may persist after acute wound healing and could trigger local foreign body reactions, making it unsuitable for short‐term treatment of acute trauma. In addition, the low melting point of raw materials may contribute to degradation of thermosensitive saponin components during electrospinning, reducing drug activity. In large‐scale production, roll‐to‐roll electrospinning may lead to uneven fibre diameter and needle clogging, complicating batch‐to‐batch quality consistency [59].

Compared with synthetic PCL fibrous membranes, chitosan/gelatin electrospun membranes offer greater hydrophilicity and improved cell adhesion and proliferation and may better simulate biological functions of the ECM. They can also retain the antibacterial and repair‐promoting activity of chitosan, with stronger synergistic effects with PNS. Their main disadvantages include poor spinnability that often requires blending with synthetic polymers to produce continuous fibres, low mechanical strength and flexibility, and a tendency to swell and collapse in moist wound environments, which compromises network structure. Their controlled‐release performance is generally inferior to that of PCL membranes.

Regarding clinical translation feasibility, PCL‐based electrospun membranes combine breathability and barrier properties of traditional gauze with repair‐promoting and controlled‐release functions of newer dressings. They can conform to wounds at highly mobile sites such as the face and joints and may be applicable to burns, infected wounds and chronic refractory wounds. For large‐scale production, electrospinning technology supports roll‐to‐roll manufacturing, and supply chains for key raw materials are established. Priorities for PCL‐based membranes include optimizing electrospinning to reduce degradation of active PNS components and establishing automated process control to improve batch consistency [59].

#### 3.2.4 Intelligent Responsive Multifunctional Dressings

Intelligent responsive multifunctional dressings are novel dressings that can sense changes in physicochemical indicators in the wound microenvironment or respond to external physical stimuli to achieve on‐demand, precise PNS release. They represent an emerging direction in wound dressing development. The most extensively studied types are pH‐responsive and near‐infrared (NIR)‐responsive PNS dressings, which differ in response mechanisms, material matrices, controlled‐release precision and clinically relevant scenarios.

pH‐responsive PNS dressings are commonly prepared by solution casting/crosslinking using pH‐sensitive polymers such as carboxymethyl chitosan and polyacrylic acid. They can respond to elevated pH during inflammatory stages by increasing drug release and slow release as pH normalizes during later healing [60]. A key advantage of such dressings lies in that their response mechanism entirely relies on the pathological microenvironment of the wound itself without external manual intervention, leading to extremely high administration convenience, especially suitable for scenarios lacking guidance from professional medical staff such as primary medical institutions and home care. Meanwhile, the pH‐responsiveness may align with healing dynamics: more severe inflammation and higher pH can trigger faster drug release, potentially meeting stage‐specific medication demands of different wound healing stages and while reducing ineffective drug release and excessive exposure. In addition, many matrices are approved medical excipients, such as carboxymethyl chitosan, supporting biosafety and compatibility with the wound microenvironment. However, these dressings have limitations. Response sensitivity and specificity can be insufficient because pH ranges vary substantially across wounds, and pH in diabetic foot and infected wounds can fluctuate significantly affected by exudate and bacterial infection, making it difficult to reproduce ideal laboratory responsiveness in complex clinical settings. In addition, responsiveness is often irreversible: once drug release is triggered by elevated wound pH, it cannot be readily stopped as healing progresses, resulting in insufficient controlled‐release flexibility. Swelling behaviour can also be affected by exudate, increasing risk of non‐specific leakage and reducing controlled‐release precision [60, 61].

NIR‐responsive nanofiber dressings typically incorporate NIR‐sensitive materials such as graphene and gold nanoparticles and use PCL/PLGA electrospun nanofibers as matrices, prepared via electrospinning combined with nanocomposite technology. These dressings can trigger PNS release through external NIR irradiation, enabling control of administration timing and dosage [59]. They provide high controlled‐release precision, enabling ‘on–off’ pulsed release by adjusting irradiation power and duration and supporting on‐demand administration aligned with changing medication needs. They can also show reversible, repeatable responsiveness, allowing multiple triggers of drug release according to wound healing status without the irreversible drug release defect of pH‐responsive dressings. Meanwhile, the photothermal effects of NIR irradiation may help reduce bacterial burden and improve local circulation, forming a synergy with the repair‐promoting effects of PNS and supporting use in chronic refractory wounds infected with drug‐resistant bacteria. However, these dressings require specialized irradiation equipment, increasing operational complexity and poor adaptability to home care and primary scenarios. Meanwhile, the NIR photothermal effects can increase local wound temperature, and inadequate temperature control may cause secondary thermal injury. In addition, long‐term in vivo biosafety and accumulation risks of materials such as graphene and gold nanoparticles remain incompletely verified, increasing regulatory thresholds for translation. High raw material costs, complex preparation, challenges in large‐scale production and higher product prices than conventional dressings further limit application [57, 59].

Based on response characteristics and performance differences, pH‐responsive dressings may be more suitable for routine treatment of conventional acute trauma and inflammatory wounds, with broader clinical application scenarios. NIR‐responsive dressings are more appropriate for complex cases requiring precise, intermittent administration, such as chronic wounds infected with drug‐resistant bacteria and scar repair. However, the clinical translation of both types remains early‐stage, with key barriers including limited stability of responsiveness in complex human wound microenvironments and the need for further verification of long‐term biosafety of responsive materials. Regarding large‐scale production, both approaches rely on industrially established technologies. pH‐responsive dressings generally have lower mass‐production thresholds, whereas NIR‐responsive systems require attention to raw material supply, cost control and batch stability [57].

#### 3.2.5 Core Issues in Clinical Translation of Novel Delivery Systems

Although these delivery systems address key limitations of traditional PNS preparations at the material science level, barriers to clinical translation remain concentrated in three core are: implementation of clinical feasibility, large‐scale production and compliance with regulatory requirements. To optimize clinical adaptability, development should move beyond material innovation alone, use clinical needs as the primary guide, and design dosage forms tailored to scenarios such as acute trauma, chronic refractory wounds, burns and scar repair. Pharmacokinetic and toxicokinetic studies in damaged skin are needed to clarify in vivo accumulation risks of nanocarriers [55, 61]. Head‐to‐head studies against first‐line clinical regimens are also needed to define core clinical advantages [41]. For large‐scale production, a standardized production and quality control system should be established. The origin and extraction process of PNS raw materials should be standardized, and unified quality control standards should be formulated [62, 63]. Scaled‐up production processes should be optimized, standardized operating procedures developed and batch inconsistency during scale‐up addressed [64]. Use of compliant domestic excipients should be prioritized to reduce cost and improve efficiency through process simplification, thereby improving clinical accessibility. For regulatory compliance, product classification and application pathways should be clarified in the early stage of research and development [8], and a full life‐cycle quality control from raw materials to finished products should be established [57]. Consistent with regulatory principles for new traditional Chinese medicines, compliant clinical trials should be conducted to provide sufficient evidence for registration [41, 42, 63].

Taken together, novel delivery systems, including PLGA/chitosan nanocarriers, hydrogels, electrospun fibrous membranes and responsive multifunctional dressings, have addressed key limitations of traditional PNS dosage forms at the materials level, such as low bioavailability, poor targeting and uneven drug release [49, 50]. These systems have significantly improved the controlled‐release performance and wound adaptability, providing technical support for PNS applications in various skin wounds. The implementation of clinical feasibility, large‐scale production capacity and regulatory compliance are the three major requirements for moving these systems from laboratory research to clinical use. Future dosage form innovation should be guided by clinical needs rather than material optimization alone and should integrate pharmacodynamic, safety, manufacturing and compliance studies to advance material innovation and clinical translation in parallel and support standardized clinical application of novel PNS preparations in wound repair (Table 2).

**TABLE 2:** Summary of properties, advantages and disadvantages of core carrier materials for *Panax notoginseng* saponins (PNS) delivery.

Carrier material type	Core matrix	Key advantages	Key disadvantages	Optimal applicable wound scenarios	Adaptability to PNS delivery	References
Nanocarriers	PLGA	Precisely controllable degradation rate, good chemical stability, steady drug release, mature regulatory compliance	Acidic degradation products may aggravate inflammation, no biological activity, high cost, complex preparation process	Chronic refractory wounds, scar repair	Suitable for long‐term encapsulation and delivery of lipophilic saponin monomers	[9, 51]
Nanocarriers	Chitosan	Natural antibacterial and hemostatic properties, strong bioadhesion, low cost, simple preparation process, excellent biocompatibility	Poor water solubility, obvious burst release effect, low mechanical strength, easy aggregation in neutral environment	Acute hemorrhagic trauma, superficial infected wounds	Improves skin penetration of PNS and forms synergistic effects of antibacterial activity and repair promotion with PNS	[52, 53]
Hydrogels	Chitosan‐based	Natural bioactivity, moisturizing and exudate absorption, antibacterial and procoagulant effects, favourable biocompatibility, mature preparation process	Poor mechanical properties, insufficient fitting to irregular wounds, weak controlled‐release performance in high‐exudate environments	Conventional wounds including acute trauma, superficial burns and pressure ulcers	Stably encapsulates PNS through its three‐dimensional structure and synergistically promotes wound repair	[54, 55]
Hydrogels	PNIPAM thermosensitive type	In situ gelation, good fitting to irregular wounds, tunable mechanical properties, precise controlled release, high patient compliance	No natural biological activity, potential toxicity of residual monomers, high cost, strict requirements for sterilization process	Lacunar wounds, sinuses, irregular wounds at joint sites	Liquid administration enables uniform dispersion of PNS, with steady drug release after gelation triggered by body temperature	[56, 57]
Electrospun fibrous membranes	PCL	Excellent spinnability, favourable mechanical properties, high porosity, long‐acting drug release, mature regulatory compliance	Strong hydrophobicity, excessively long degradation cycle, easy degradation of PNS activity during electrospinning	Wounds at joint sites, burns, chronic refractory wounds, high infection‐risk wounds	Fibrous network structure enables uniform loading and ultra‐long‐acting steady release of PNS	[58, 59]
Intelligent responsive dressings	pH‐sensitive polymers	No external intervention required, convenient administration, response characteristics matching healing process, good biosafety	Insufficient response sensitivity, irreversible drug release, prone to non‐specific leakage affected by exudate	Conventional acute trauma, wounds in inflammatory stage, primary medical/home care scenarios	Rapid release of PNS in the inflammatory stage and slow release in the late healing stage, precisely matching medication requirements	[60, 61]
Intelligent responsive dressings	NIR‐sensitive materials	Extremely high controlled‐release precision, reversible and repeatable drug release, photothermal effect for synergistic antibacterial and anti‐infective activity	Requires professional equipment, risk of thermal injury, safety of functional materials to be verified, high cost	Chronic wounds infected with drug‐resistant bacteria, scar repair and other scenarios requiring precise intermittent administration	Pulsatile on‐demand release of PNS realized by external irradiation, with precise regulation of dosage and timing	[57, 59]

## 4 Research Challenges and Future Perspectives

Although numerous basic studies and preliminary clinical explorations support the pharmacological mechanisms and application potential of PNSs in skin wound repair, several challenges remain, including limited mechanistic precision, incomplete translation of dosage forms and insufficient clinical evidence. Addressing these limitations and defining future research priorities are essential to promote the transition of PNSs from basic research to standardized clinical application.

### 4.1 Current Research Limitations

#### 4.1.1 Insufficient Precision in Mechanistic Research

The wound‐healing efficacy of PNSs, a multicomponent natural mixture, depends on synergistic actions among monomeric saponins. However, the specific molecular targets of key active components have not been fully defined. Most studies focus on modulatory effects of a single component or individual signalling pathways, whereas systematic analysis of synergistic or antagonistic relationships among components remains limited [51]. In addition, pathways modulated by PNSs in wound repair—including NF‐κB, PI3K/AKT and TGF‐β1/Smad—form a complex interaction network, and mechanisms underlying pathway crosstalk are not fully understood. Thus, identifying key molecular nodes through which PNSs exert effects remains a major challenge [53]. Moreover, many traditional cellular and animal models do not replicate the complexity of the human wound microenvironment. Accordingly, the clinical relevance of mechanistic findings requires further strengthening [64].

Another critical limitation regarding mechanistic precision is the lack of direct biophysical validation of protein‐ligand interactions. Current evidence for the targeting of PI3K and TGF‐β1 receptors by ginsenoside Rg1 and Rb1 relies predominantly on pharmacological functional assays, such as detection of downstream phosphorylation, reporter gene activity and gene overexpression/knockdown experiments. Furthermore, several of these target‐related conclusions are extrapolated from disease models other than skin wound repair. To date, no study has employed chemoproteomic target deconvolution approaches‐such as surface plasmon resonance (SPR), microscale thermophoresis (MST), cellular thermal shift assay (CETSA) or target fishing coupled with mass spectrometry‐to systematically identify the direct binding targets of PNS monomers in skin‐wound‐relevant systems. This gap prevents definitive confirmation of direct molecular targets and limits the precision of mechanistic interpretation.

#### 4.1.2 Dosage Form Optimization

Although novel delivery systems address some shortcomings of traditional dosage forms, several issues remain unresolved. Some nanocarriers lack long‐term in vivo metabolic data, and their biocompatibility at wound sites, toxicity of degradation products, and long‐term accumulation risks have not been fully verified [55]. Because the moist wound microenvironment is rich in proteolytic enzymes, nanocarriers may rupture and release drugs prematurely, compromising formulation stability and controlled release [65]. In addition, development of intelligent dressings remains restricted to the laboratory stage. The complexity of their preparation processes, high raw material costs, and potential batch variation during large‐scale production make it difficult to meet requirements of large‐scale clinical application [57]. Furthermore, most existing dosage forms are designed for a single repair need, whereas whole‐process treatment of complex wounds requires multifunctional integrated formulations that combine anti‐infection, pro‐repair and anti‐scarring properties with healing status monitoring [61].

#### 4.1.3 Gaps in Clinical Research

Existing clinical data are insufficient to support standardized clinical application of PNSs. A primary limitation is study scale: most published clinical studies are single‐centre, small‐sample investigations that lack the rigour of large, multicentre randomized controlled trials (RCTs). As a result, the level of evidence is limited and does not allow robust conclusions regarding efficacy and safety. Moreover, most studies have short follow‐up periods, which precludes an adequate evaluation of the safety of PNSs and the recurrence rates of scars over the long‐term [41]. In addition, personalized treatment regimens are also lacking. Significant differences in pathophysiological characteristics exist between different wound types and healing stages, yet no personalized administration dosages, treatment courses or combined schemes have been established for specific wound depths or patient ages. Therefore, clinical use often relies on empirical prescribing [66]. Finally, quality control standards for topical PNS formulations have not been fully unified, and differences in purity and active component proportions across manufacturers hinder consistency and comparability of clinical findings [62].

#### 4.1.4 Effects of Extraction Processes on the Pharmacological Efficacy of PNS and Gaps in Standardized Research

As an active mixture of multiple saponin monomers in Panax notoginseng, pharmacological activity and therapeutic efficacy of PNS in skin wound repair are determined by the composition and relative proportions of active ingredients. Different extraction and purification processes influence yield, content and ratios of key monomers, product purity and impurity residues, and can therefore directly affect therapeutic efficacy [8, 62]. Current preparation techniques for PNS include traditional ethanol reflux, ultrasonic‐assisted extraction, microwave‐assisted extraction, enzymatic‐assisted extraction and supercritical CO_2_ extraction, among others. These methods differ in mechanisms and applicable scenarios. For instance, ultrasonic‐assisted extraction enhances cell membrane permeability via cavitation, greatly improving the dissolution rate of key monomers such as ginsenosides Rg1 and Rb1; the proportion of these two monomers in the final product may be higher than that obtained using traditional ethanol reflux [51, 53]. Supercritical CO_2_ extraction enables efficient isolation of PNS under low‐temperature and oxygen‐free conditions, substantially reducing impurities such as polysaccharides and heavy metals and improving product purity. However, its extraction efficiency for the highly polar notoginsenoside R1 is significantly lower than that achieved with alcohol extraction [8]. Enzymatic‐assisted extraction uses cellulase and pectinase to degrade the plant cell wall, increasing total saponin yield, but inadequate control of enzymatic conditions may degrade some active saponin monomers [53]. Such process differences can lead to substantial variation in the Rg1:Rb1 ratio—the key monomers underlying wound repair efficacy—in PNS from different sources. Because these two monomers target different stages of skin wound repair, changes in their ratio can alter the intensity of core pharmacological effects, including anti‐inflammatory, promotion of epithelialization, angiogenesis and anti‐scarring effects [9, 10, 18, 19], and may even reverse the direction of their synergistic or antagonistic interactions [36]. This may help explain why equivalent doses of PNS show divergent therapeutic effects across skin wound repair models, with some conclusions appearing contradictory [17, 19]. However, most basic and clinical studies report only PNS dose and total saponin purity, without specifying the extraction and purification parameters or precise monomer content ratios, limiting reproducibility and comparability of findings [62]. Furthermore, PNS raw materials from different manufacturers can show substantial batch‐to‐batch variation because of nonstandardized processes. This not only fails to meet raw material quality control requirements for drug and medical device registration [63], but also restricts standardized clinical application and translation of PNS in skin wound repair.

### 4.2 Future Research Directions

#### 4.2.1 In‐Depth Analysis of Molecular Mechanisms

Improving the precision and systematic nature of mechanistic research will require integration of cutting‐edge biotechnology. Single‐cell sequencing and spatial transcriptomics may be used to identify specific targets of key monomeric saponins in *Panax notoginseng*, such as Rg1 and Rb1, across wound repair stages. These high‐resolution methods can help distinguish synergistic and independent modulatory effects of different components [67]. In addition, proteomics and metabolomics should be used to analyse crosstalk within NF‐κB, PI3K/AKT and TGF‐β1/Smad pathways, facilitating construction of molecular network maps of PNS‐modulated wound repair and identification of key regulatory nodes [68]. Concurrently, development of more clinically relevant disease models is needed to better simulate the complex wound microenvironment and improve clinical relevance of mechanistic findings [69].

#### 4.2.2 Upgrading and Breakthroughs in Dosage Form Innovation

Future dosage form optimization should prioritize three goals: precise delivery, multifunctional integration and clinical adaptability. A key direction is development of pH/temperature dual‐responsive intelligent dressings designed to trigger drug release through dual stimulation from the wound microenvironment, supporting rapid release during the inflammatory phase, slower controlled release during proliferation and on‐demand release during remodelling [61]. Another direction is integration of 3D bioprinting to prepare tissue‐engineered skin using biocompatible carriers loaded with PNSs and seeded cells for treatment of refractory wounds such as large‐area burns and chronic ulcers [70]. This approach aims to support synergy among scaffold support, drug regulation and cell regeneration. In addition, research should prioritize surface modification of nanocarriers with cell‐penetrating peptides and targeting molecules to enhance skin penetration while minimizing systemic accumulation [55]. Finally, improving the stability of these formulations in moist wound environments remains for extending the duration of drug action [59].

#### 4.2.3 Standardized Advancement of Clinical Translation

A primary objective is to strengthen clinical research design and facilitate the transformation of research achievements. The field should prioritize multicentre, large‐sample RCTs with long‐term follow‐up that include diverse wound types and age strata. These trials can systematically verify efficacy and long‐term safety of PNS formulations and provide higher‐level evidence to guide clinical use [42]. Development of personalized regimens is also needed. Clinical data should be used to establish differentiated dosages, treatment courses and combination strategies based on wound depth, patient comorbidities and healing stage [71]. Standardizing quality control for topical PNS formulations is equally essential, including unified standards for raw material purity, active component proportions, heavy metal residues and microbial limits to ensure product consistency. In parallel, industrialization should be accelerated by optimizing processes to reduce costs of intelligent dressings and increasing manufacturing capacity to meet clinical demand [63].

#### 4.2.4 Innovative Exploration of Interdisciplinary Integration

A key future direction is to extend application boundaries through interdisciplinary collaboration. One target is development of multifunctional dressings that integrate diagnostic, therapeutic and monitoring capabilities through convergence of materials science, nanotechnology and biomedical engineering. In addition, integration of pH sensors and inflammatory factor detection modules should be integrated to allow real‐time monitoring of wound healing status. Such systems could transmit data to terminal devices via wireless signals and automatically adjust PNS release dosage, supporting precise, intelligent treatment [72]. Exploring the combined application of PNSs with stem cell and gene therapy also remains a priority. Co‐delivery of PNSs with mesenchymal stem cells (MSCs) may improve the survival microenvironment and repair capacity of the cells. Additionally, the construction of composite carriers loaded with both PNSs and therapeutic genes aim to support synergistic ‘drug regulation + gene intervention’ strategies for refractory wounds [73]. Finally, research into the synergistic effects between PNSs and other natural active components is needed to develop multicomponent preparations that utilize multitarget mechanisms for comprehensive repair [58] (Table 3).

**TABLE 3:** Hierarchical summary of research evidence on *Panax notoginseng* saponins in skin wound repair.

Evidence level	Research type	Core research module	Key research findings	Explanation of evidence‐based medicine level	References
Basic research evidence	In vitro cellular experiments	Inflammation and immune regulation	*Panax notoginseng* saponins (PNS) can significantly downregulate the transcription and expression of pro‐inflammatory cytokines including IL‐6, IL‐8 and TNF‐α in macrophages and wound tissues, thus reducing inflammatory cell infiltration It can block the NF‐κB pathway by inhibiting IκBα degradation, and simultaneously suppress the phosphorylation of ERK1/2, p38 and JNK in the MAPK pathway, thereby interrupting the inflammatory cascade It can upregulate the expression of the anti‐inflammatory cytokine IL‐10, induce macrophage polarization from the pro‐inflammatory M1 phenotype to the reparative M2 phenotype, and promote the transition from the inflammatory phase to the proliferative phase	Level 4 Evidence (In vitro basic experimental evidence that provides cellular support for mechanistic studies, with the lowest evidentiary strength. It can only be used for preliminary verification of efficacy and mechanisms and cannot be directly extrapolated to in vivo or clinical settings)	[14, 16, 18, 19, 20]
Basic research evidence	In vitro cellular experiments	Regulation of cellular functions in the proliferative phase	PNS can activate the PI3K/AKT‐ERK1/2 pathway, promote the transition of HaCaT keratinocytes from the G0/G1 phase to the S phase, enhance cell proliferation activity and upregulate MMP‐2/9 activity to strengthen cell migration, thereby accelerating the epithelialization process It can regulate the expression of TGF‐β1 receptors in fibroblasts, promote the synthesis of collagen type I/III and maintain their physiological ratio of 3:1, providing stable extracellular matrix (ECM) support for the wound It exerts a bidirectional regulatory effect on fibroblast proliferation: it activates normal fibroblasts to fill the wound, while inhibiting the excessive proliferation of scar‐forming fibroblasts	Level 4 Evidence (In vitro basic experimental evidence that clarifies the regulatory effects of PNS on core wound repair cells, laying a mechanistic foundation for in vivo experiments)	[27, 28, 29, 30, 31]
Basic research evidence	In vitro cellular experiments	Angiogenesis regulation	PNS can significantly upregulate the transcription and secretion of VEGF and bFGF in HUVECs, which synergistically mediate endothelial cell proliferation, migration and tubular structure formation It can enhance endothelial cell proliferation and anti‐apoptotic ability and promote vascular lumen formation by activating the JAK2‐STAT3 and PI3K/AKT pathways It can inhibit platelet adhesion and aggregation, improve coagulation function and provide a favourable microenvironment for angiogenesis	Level 4 Evidence (In vitro basic experimental evidence that elucidates the cellular mechanisms underlying the pro‐angiogenic effect of PNS, providing a theoretical basis for verifying angiogenesis in vivo)	[32, 33, 34]
Basic research evidence	In vitro cellular experiments	Remodelling phase and scar inhibition regulation	PNS can bidirectionally regulate collagen metabolism: it not only promotes the orderly synthesis of collagen by fibroblasts but also inhibits the abnormal deposition of collagen fibres. Meanwhile, it regulates the balance of MMPs/TIMP‐1/2 to maintain the homeostasis of ECM synthesis and degradation It can block the TGF‐β1/Smad2/3 pathway, inhibit the transformation of fibroblasts into myofibroblasts, downregulate α‐SMA expression and reduce scar contraction It can increase the activities of SOD and GSH‐Px, decrease the level of MDA, scavenge reactive oxygen species (ROS) and alleviate oxidative stress damage to repair cells	Level 4 Evidence (In vitro basic experimental evidence that clarifies the cellular mechanisms of anti‐scarring and anti‐oxidative stress effects of PNS, providing support for in vivo scar repair experiments)	[9, 10, 27, 29, 38, 39, 40]
Preclinical research evidence	In vivo animal experiments	Efficacy verification in different types of trauma	Acute trauma: PNS cream can shorten the healing time of incisional wounds on the back of rats, improve wound tensile strength and reduce the risk of infection Chronic refractory wounds: PNS composite hydrogel can increase the wound healing rate in rat models of diabetic foot ulcers and pressure ulcers, ameliorate repair cell dysfunction caused by hyperglycemia, and increase the number of newborn hair follicles and sebaceous glands Burns: PNS ointment preparations can upregulate the expression of PDGF‐BB/PDGFR‐β in superficial second‐degree burn wounds in rats, accelerate granulation tissue hyperplasia and epithelial repair and shorten scab shedding time Scar repair: PNS preparations can reduce scar area in mice, decrease scar tissue hardness and contractility, with no obvious local adverse reactions	Level 3 Evidence (Preclinical in vivo animal evidence that validates the overall efficacy and safety of PNS in living wounds, serving as a key bridge for the translation from in vitro studies to clinical applications, with higher evidentiary strength than in vitro cellular experiments)	[9, 10, 32, 42, 44, 45, 46, 47, 48]
Preclinical research evidence	In vivo animal experiments	In vivo mechanism and efficacy verification	Inflammation regulation: In vivo studies confirm that PNS can inhibit the activation of NF‐κB and MAPK pathways in wounds, reduce the release of pro‐inflammatory cytokines, induce M2 macrophage polarization and balance the wound immune microenvironment Angiogenesis: PNS can significantly increase microvessel density (MVD) and the proportion of CD34‐positive cells in wound tissues of rat diabetic foot ulcer models, and improve local blood perfusion Anti‐oxidative stress: In vivo studies verify that PNS can elevate the activities of SOD and GSH‐Px in wound tissues, decrease MDA levels and alleviate oxidative stress injury	Level 3 Evidence (Preclinical in vivo animal evidence that validates the molecular mechanisms of PNS in regulating wound repair at the living organism level, providing more physiologically relevant support for mechanistic conclusions)	[21, 32, 33, 34, 35, 39, 40]
Preclinical research evidence	In vivo animal experiments	Efficacy verification of novel delivery systems	PLGA/chitosan nanoparticles can improve the biostability, skin penetration and local bioavailability of PNS and achieve sustained drug release Chitosan/PNS composite hydrogels and thermosensitive hydrogels can adapt to the wound microenvironment, with functions of moisturizing, scaffold support and controlled drug release, significantly improving wound healing quality Electrospun fibrous membranes can simulate the ECM structure, guide ordered cell growth, and construct a synergistic system of ‘repair + anti‐infection’ pH/NIR‐responsive dressings can realize intelligent on‐demand release of PNS, precisely match the time sequence of wound healing, and enhance drug utilization efficiency	Level 3 Evidence (Preclinical in vivo animal evidence that validates the practical application effects of different delivery systems, providing core data support for the clinical translation of PNS dosage forms)	[9, 46, 52, 54, 56, 57, 58, 59, 60]
Clinical application evidence	Human clinical studies	Exploration of clinical efficacy and safety	Acute trauma/postoperative incisions: Topical PNS preparations can accelerate the healing of surgical incisions and acute traumatic wounds, reduce the risk of postoperative infection, and provide a new strategy for clinical wound care Burn wounds: PNS preparations can promote scab shedding and epithelial repair in patients with superficial second‐degree burns, improve wound healing rates and shorten the treatment course Chronic refractory wounds: PNS composite preparations show promising healing‐promoting potential in patients with diabetic foot ulcers and pressure ulcers, which can shorten the healing period and improve patients' quality of life Safety: Topical administration of PNS exhibits no obvious local irritation, allergy or other adverse reactions in clinical practice, with favourable skin safety and biocompatibility	Level 2 Evidence (Evidence from single‐centre, small‐sample exploratory clinical studies, providing preliminary human data to support the clinical application of PNS, with higher evidentiary strength than preclinical animal experiments. However, limited by sample size and study design, it has a low grade in evidence‐based medicine and cannot support high‐level clinical recommendations)	[44, 47, 48, 62, 66]
Clinical application evidence	Summary of clinical research status	Gaps in clinical evidence	At present, there is a lack of multicentre, large‐sample, randomized controlled clinical trials (RCTs) with long‐term follow‐up, and no high‐level evidence‐based medical evidence supports the standardized clinical application of PNS. Meanwhile, individualized administration regimens for different trauma types and healing stages, as well as unified quality control standards for topical PNS preparations, are still insufficient	Level 1 Evidence Gap (Multicentre, large‐sample RCTs represent the highest Level 1 evidence in evidence‐based medicine, serving as the core support for the standardized clinical application of PNS and also the key gap in current research)	[41, 42, 62, 66]

## 5 Conclusions

Skin wound repair relies on coordinated crosstalk among inflammation regulation, cell proliferation, angiogenesis and tissue remodelling. Refractory wounds, characterized by microenvironmental imbalance and disordered repair mechanisms, continue to pose challenges of low healing efficiency, high complication rates and limited clinical strategies. As a natural multicomponent active substance, PNS, with ginsenosides Rg1 and Rb1 as key monomers, regulate each wound repair stage through multitarget and multipathway synergistic effects. PNS balances the immune microenvironment and suppresses excessive inflammation during the inflammatory phase; accelerates epithelialization and granulation tissue formation during the proliferative phase; improves perfusion and promotes microvascular development during angiogenesis; and maintains collagen metabolic homeostasis while inhibiting excessive scar hyperplasia during remodelling, thereby promoting wound healing and improving healing quality. Extraction and purification processes determine the ratio and purity of key monomers in PNS and thereby influence wound repair efficacy. Novel delivery systems, including PLGA, chitosan, hydrogels, electrospun fibres and intelligent responsive materials, have improved local retention, controlled‐release performance, and wound compatibility of PNS, supporting clinical translation. However, current research still has limitations, including imprecise mechanistic characterization, unclear synergistic and antagonistic interactions among monomers, insufficient formulation stability and scalability for mass production, limited high‐quality clinical evidence, and inconsistent raw material processing and quality control standards. Future studies should focus on molecular targets and synergistic mechanisms of key monomers, standardization of extraction processes, optimization of intelligent delivery systems, and multicentre, large‐sample RCTs to improve targeting efficiency, stability and clinical applicability of PNS. In summary, PNS has well‐defined effects, unique advantages and broad application prospects in skin wound repair. With deeper mechanistic research, innovative dosage form development and standardized clinical studies, PNS may become an important therapeutic approach for refractory wounds and support modernized, precise translation of active ingredients from traditional Chinese medicine.

## Author Contributions

**Zhifeng Wang:** conceptualization, methodology, software, data curation, investigation, validation, formal analysis, supervision, writing – original draft. **Ting Shi:** conceptualization, writing – original draft, methodology. **Daman Tian:** conceptualization, validation, software. **Rui Li:** validation, formal analysis. **Ting Yao:** software, data curation. **Junfeng Lan:** investigation, validation. **Yujiang Xi:** writing – review and editing. **RuiRui Wang:** writing – review and editing, funding acquisition. **Jian Wang:** writing – review and editing, funding acquisition.

## Funding

This project was supported by the National Natural Science Foundation of China (No. 82560932), the Major Project of the Joint Foundation of Yunnan University of Traditional Chinese Medicine (No. XYLH2024001), the General Project of Applied Basic Research of Yunnan Province (No. 202201AT070214), and the Two Talents Program of Yunnan Province (No. 202205AD160024), the Joint Special Fund for Basic Research of Traditional Chinese Medicine Application of the Department of Science and Technology of Yunnan Province (No. 202001AZ070001‐020, 202301AZ070001‐016), the Scientific Research Support Project of the Department of Education of Yunnan Province (No. 2025Y0632) and the Academician‐Expert Workstation of Yunnan Province (No. 202505AF350033).

## Ethics Statement

The authors have nothing to report.

## Consent

The authors have nothing to report.

## Conflicts of Interest

The authors declare no conflicts of interest.

## Data Availability

The authors have nothing to report.

## References

[1] T. A. Harris‐Tryon and E. A. Grice , “Microbiota and Maintenance of Skin Barrier Function,” Science 376, no. 6596 (2022): 940–945, 10.1126/science.abo0693. 35617415

[2] O. A. Peña and P. Martin , “Cellular and Molecular Mechanisms of Skin Wound Healing,” Nature Reviews Molecular Cell Biology 25, no. 8 (2024): 599–616, 10.1038/s41580-024-00715-1. 38528155

[3] A. A. Mamun , C. Shao , P. Geng , S. Wang , and J. Xiao , “Recent Advances in Molecular Mechanisms of Skin Wound Healing and Its Treatments,” Frontiers in Immunology 15 (2024): 1395479, 10.3389/fimmu.2024.1395479. PMC11148235 38835782

[4] N. Graves , C. J. Phillips , and K. Harding , “A Narrative Review of the Epidemiology and Economics of Chronic Wounds,” British Journal of Dermatology 187, no. 2 (2022): 141–148, 10.1111/bjd.20692. 34549421

[5] M. S. Conte , A. W. Bradbury , P. Kolh , et al., “Global Vascular Guidelines on the Management of Chronic Limb‐Threatening Ischemia,” Journal of Vascular Surgery 69, no. 6S (2019): 3S–125S.e40, 10.1016/j.jvs.2019.02.016. PMC8365864 31159978

[6] M. Olsson , K. Järbrink , U. Divakar , et al., “The Humanistic and Economic Burden of Chronic Wounds: A Systematic Review,” Wound Repair and Regeneration 27, no. 1 (2019): 114–125, 10.1111/wrr.12683. 30362646

[7] L. Teot and N. Ohura , “Challenges and Management in Wound Care,” Plastic and Reconstructive Surgery 147, no. 1S‐1 (2021): 9S–15S, 10.1097/PRS.0000000000007628. 33347058

[8] Y. Xie and C. Wang , “Herb‐Drug Interactions Between *Panax notoginseng* or Its Biologically Active Compounds and Therapeutic Drugs: A Comprehensive Pharmacodynamic and Pharmacokinetic Review,” Journal of Ethnopharmacology 307 (2023): 116156, 10.1016/j.jep.2023.116156. 36754189

[9] S. Y. Men , Q. L. Huo , L. Shi , et al., “ *Panax notoginseng* Saponins Promotes Cutaneous Wound Healing and Suppresses Scar Formation in Mice,” Journal of Cosmetic Dermatology 19, no. 2 (2020): 529–534, 10.1111/jocd.13042. 31267657

[10] Y. Zhi , H. Wang , B. Huang , et al., “ *Panax notoginseng* Saponins Suppresses TRPM7 via the PI3K/AKT Pathway to Inhibit Hypertrophic Scar Formation In Vitro,” Burns 47, no. 4 (2021): 894–905, 10.1016/j.burns.2020.10.003. 33143990

[11] B. K. Sun , Z. Siprashvili , and P. A. Khavari , “Advances in Skin Grafting and Treatment of Cutaneous Wounds,” Science 346, no. 6212 (2014): 941–945, 10.1126/science.1253836. 25414301

[12] J. Gao , M. Yao , Y. Zhang , Y. Jiang , and J. Liu , “ *Panax notoginseng* Saponins Stimulates the Differentiation and Neurite Development of C17.2 Neural Stem Cells Against OGD/R Injuries via mTOR Signaling,” Biomedicine and Pharmacotherapy 172 (2024): 116260, 10.1016/j.biopha.2024.116260. 38382327

[13] Z. Wang , F. Qi , H. Luo , G. Xu , and D. Wang , “Inflammatory Microenvironment of Skin Wounds,” Frontiers in Immunology 13 (2022): 789274, 10.3389/fimmu.2022.789274. PMC8920979 35300324

[14] L. Liu , J. Li , Y. Wang , et al., “Effects of *Panax notoginseng* Saponins on Alleviating Low Shear Induced Endothelial Inflammation and Thrombosis via Piezo1 Signalling,” Journal of Ethnopharmacology 335 (2024): 118639, 10.1016/j.jep.2024.118639. 39084271

[15] Q. Zheng , T. Wang , S. Wang , et al., “The Anti‐Inflammatory Effects of Saponins From Natural Herbs,” Pharmacology and Therapeutics 269 (2025): 108827, 10.1016/j.pharmthera.2025.108827. 40015518

[16] S. Hu , T. Liu , Y. Wu , et al., “ *Panax notoginseng* Saponins Suppress Lipopolysaccharide‐Induced Barrier Disruption and Monocyte Adhesion on bEnd.3 Cells via the Opposite Modulation of Nrf2 Antioxidant and NF‐κB Inflammatory Pathways,” Phytotherapy Research 33, no. 12 (2019): 3163–3176, 10.1002/ptr.6488. 31468630

[17] K. D. Tsai , S. M. Yang , J. C. Lee , et al., “ *Panax notoginseng* Attenuates Bleomycin‐Induced Pulmonary Fibrosis in Mice,” Evidence‐Based Complementary and Alternative Medicine 2011 (2011): 404761, 10.1155/2011/404761. PMC3057514 21423633

[18] Y. Wang , X. Sun , Y. Xie , et al., “ *Panax notoginseng* Saponins Alleviate Diabetic Retinopathy by Inhibiting Retinal Inflammation: Association With the NF‐κB Signaling Pathway,” Journal of Ethnopharmacology 319, no. Pt 1 (2024): 117135, 10.1016/j.jep.2023.117135. 37689326

[19] H. Luo , C. T. Vong , D. Tan , et al., “ *Panax notoginseng* Saponins Modulate the Inflammatory Response and Improve IBD‐Like Symptoms via TLR/NF‐κB and MAPK Signaling Pathways,” American Journal of Chinese Medicine 49, no. 4 (2021): 925–939, 10.1142/S0192415X21500440. 33829964

[20] B. Kim , E. Y. Kim , E. J. Lee , et al., “ *Panax notoginseng* Inhibits Tumor Growth Through Activating Macrophage to M1 Polarization,” American Journal of Chinese Medicine 46, no. 6 (2018): 1369–1385, 10.1142/S0192415X18500726. 30168347

[21] M. Halle , A. Gabrielsen , G. Paulsson‐Berne , et al., “Sustained Inflammation due to Nuclear Factor‐Kappa B Activation in Irradiated Human Arteries,” Journal of the American College of Cardiology 55, no. 12 (2010): 1227–1236, 10.1016/j.jacc.2009.10.047. 20298930

[22] L. J. Egan , A. de Lecea , E. D. Lehrman , G. M. Myhre , L. Eckmann , and M. F. Kagnoff , “Nuclear Factor‐Kappa B Activation Promotes Restitution of Wounded Intestinal Epithelial Monolayers,” American Journal of Physiology Cell Physiology 285, no. 5 (2003): C1028–C1035, 10.1152/ajpcell.00167.2003. 12826601

[23] J. W. Christman , L. H. Lancaster , and T. S. Blackwell , “Nuclear Factor Kappa B: A Pivotal Role in the Systemic Inflammatory Response Syndrome and New Target for Therapy,” Intensive Care Medicine 24, no. 11 (1998): 1131–1138, 10.1007/s001340050735. PMC7094907 9876974

[24] X. Shi , W. Yu , L. Liu , et al., “ *Panax notoginseng* Saponins Administration Modulates Pro‐/Anti‐Inflammatory Factor Expression and Improves Neurologic Outcome Following Permanent MCAO in Rats,” Metabolic Brain Disease 32, no. 1 (2017): 221–233, 10.1007/s11011-016-9901-3. 27585466

[25] L. Zhao , S. Tang , F. Chen , X. Ren , X. Han , and X. Zhou , “Regulation of Macrophage Polarization by Targeted Metabolic Reprogramming for the Treatment of Lupus Nephritis,” Molecular Medicine 30, no. 1 (2024): 96, 10.1186/s10020-024-00866-z. PMC11197188 38914953

[26] D. L. Laskin , “Macrophages and Inflammatory Mediators in Chemical Toxicity: A Battle of Forces,” Chemical Research in Toxicology 22, no. 8 (2009): 1376–1385, 10.1021/tx900086v. PMC2787782 19645497

[27] J. P. Dai , X. X. Chen , D. X. Zhu , et al., “ *Panax notoginseng* Saponins Inhibit Areca Nut Extract‐Induced Oral Submucous Fibrosis In Vitro,” Journal of Oral Pathology & Medicine 43, no. 6 (2014): 464–470, 10.1111/jop.12158. 24484214

[28] J. Chéret , N. Lebonvallet , V. Buhé , J. L. Carre , L. Misery , and C. Le Gall‐Ianotto , “Influence of Sensory Neuropeptides on Human Cutaneous Wound Healing Process,” Journal of Dermatological Science 74, no. 3 (2014): 193–203, 10.1016/j.jdermsci.2014.02.001. 24630238

[29] V. Nguyen , F. Pan , G. Zhang , Q. Zhang , and Y. Lu , “ *Panax notoginseng* Saponins Regulate Transforming Growth Factor‐β1 Through MAPK and Snail/TWIST1 Signaling Pathway to Inhibit Epithelial‐Mesenchymal Transition of Pulmonary Fibrosis in A549 Cells,” Evidence‐Based Complementary and Alternative Medicine 2022 (2022): 3744618, 10.1155/2022/3744618. PMC9296299 35865337

[30] Y. Xu , L. Cao , W. Zou , R. Yu , and W. Shen , “ *Panax notoginseng* Saponins Inhibits NLRP3 Inflammasome‐Mediated Pyroptosis by Downregulating lncRNA‐ANRIL in Cardiorenal Syndrome Type 4,” Chinese Medicine 18, no. 1 (2023): 50, 10.1186/s13020-023-00756-2. PMC10165771 37158944

[31] X. Hu , H. Zhang , X. Li , Y. Li , and Z. Chen , “Activation of mTORC1 in Fibroblasts Accelerates Wound Healing and Induces Fibrosis in Mice,” Wound Repair and Regeneration 28, no. 1 (2020): 6–15, 10.1111/wrr.12759. 31444991

[32] T. Lei , Y. Gao , Y. Duan , C. Cui , L. Zhang , and M. Si , “ *Panax notoginseng* Saponins Improves Healing of High Glucose‐Induced Wound Through the GSK‐3β/β‐Catenin Pathway,” Environmental Toxicology 37, no. 8 (2022): 1867–1877, 10.1002/tox.23533. 35385194

[33] C. G. Wong , M. Taban , K. Osann , et al., “Subchoroidal Release of VEGF and bFGF Produces Choroidal Neovascularization in Rabbit,” Current Eye Research 42, no. 2 (2017): 237–243, 10.1080/02713683.2016.1227448. 27749103

[34] Q. Zhou , L. Jiang , C. Xu , et al., “Ginsenoside Rg1 Inhibits Platelet Activation and Arterial Thrombosis,” Thrombosis Research 133, no. 1 (2014): 57–65, 10.1016/j.thromres.2013.10.032. 24196231

[35] J. Hui , J. Gao , Y. Wang , et al., “ *Panax notoginseng* Saponins Ameliorates Experimental Hepatic Fibrosis and Hepatic Stellate Cell Proliferation by Inhibiting the Jak2/Stat3 Pathways,” Journal of Traditional Chinese Medicine 36, no. 2 (2016): 217–224, 10.1016/s0254-6272(16)30030-9. 27400477

[36] M. M. Wang , M. Xue , Z. H. Xin , et al., “ *Panax notoginseng* Saponin Attenuates Gastric Mucosal Epithelial Cell Injury Induced by Dual Antiplatelet Drugs Through COX and PI3K/Akt/VEGF‐GSK‐3β‐RhoA Network Pathway,” Chinese Journal of Integrative Medicine 27, no. 11 (2021): 819–824, 10.1007/s11655-021-2854-3. 33449280

[37] G. Shi , Z. M. Mao , Y. G. Wan , et al., “Pathomechanisms of Podocyte Injury in Diabetic Nephropathy and Interventional Effects of Chinese Herbal Medicine,” Zhongguo Zhong Yao Za Zhi 41, no. 13 (2016): 2416–2421, 10.4268/cjcmm20161308. 28905562

[38] X. H. Du , H. Y. Li , S. Wang , and X. Niu , “Protective Effects of *Panax notoginseng* Saponins on H_2_O_2_‐Induced Damage in Skin Fibroblasts,” Lishizhen Medicine and Materia Medica Research 22, no. 10 (2011): 2549–2550, 10.3969/j.issn.1008-0805.2011.10.104.

[39] Y. Fan , Y. Qiao , J. Huang , and M. Tang , “Protective Effects of *Panax notoginseng* Saponins Against High Glucose‐Induced Oxidative Injury in Rat Retinal Capillary Endothelial Cells,” Evidence‐Based Complementary and Alternative Medicine 2016 (2016): 5326382, 10.1155/2016/5326382. PMC4785264 27019662

[40] Y. Zhang , X. Chi , Z. Wang , et al., “Protective Effects of *Panax notoginseng* Saponins on PME‐Induced Nephrotoxicity in Mice,” Biomedicine and Pharmacotherapy 116 (2019): 108970, 10.1016/j.biopha.2019.108970. 31103823

[41] B. Yan , Y. Ning , J. Guo , L. Liu , and C. Wang , “Network Pharmacology Analysis and Clinical Verification of *Panax notoginseng* Saponins in Deep Venous Thrombosis Prevention,” Biomedical Reports 22, no. 1 (2024): 8, 10.3892/br.2024.1886. PMC11572032 39559819

[42] Y. L. Dai , X. D. Deng , B. H. Niu , D. M. Wang , and Y. Zhao , “The Effect of *Panax notoginseng* Saponins Composite Nanovesicles on Skin Wound Healing and Angiogenesis in Rats With Diabetic Foot Ulcers,” Journal of Guangxi Medical University 41, no. 1 (2024): 51–57, 10.16190/j.cnki.45-1211/r.2024.01.008.

[43] H. Huang , X. Yang , X. Qin , et al., “Co‐Assembled Supramolecular Hydrogel of Asiaticoside and *Panax notoginseng* Saponins for Enhanced Wound Healing,” European Journal of Pharmaceutics and Biopharmaceutics 207 (2025): 114617, 10.1016/j.ejpb.2024.114617. 39701194

[44] C. Qin , X. Y. Kong , C. X. Liao , et al., “Effects of Xuesaitong Capsules (*Panax notoginseng* Saponins) on Ischemia/Reperfusion Injury in Mice With Skin Frostbite,” Chinese Traditional Patent Medicine 47, no. 2 (2025): 430–437, 10.3969/j.issn.1001-1528.2025.02.011.

[45] Y. J. Ha , K. H. Tak , J. M. Jung , et al., “The Effect of Polynucleotide‐Hyaluronic Acid Hydrogel in the Recovery After Mechanical Skin Barrier Disruption,” Skin Research and Technology 30, no. 9 (2024): e70068, 10.1111/srt.70068. PMC11413333 39300806

[46] W. X. Lu , Preparation and Application of Mussel‐Inspired Hydrogel Loaded With Total Saponins of Panax notoginseng Flowers in Wound Healing (Shanghai University of Traditional Chinese Medicine, 2020), 10.27320/d.cnki.gszyu.2020.000054.

[47] X. X. Cui , J. W. Huang , L. F. An , W. Y. Niu , X. Han , and X. Zhang , “Research Progress of *Panax notoginseng* (Traditional Chinese Medicine) and Its Active Components in Promoting Skin Wound Healing,” Journal of Modern Integrated Traditional Chinese and Western Medicine 32, no. 21 (2023): 3038–3043, 10.3969/j.issn.1008-8849.2023.21.022.

[48] L. Zhang , J. Y. Yang , Y. Yang , et al., “ *Panax notoginseng* Saponins Regulates the Expression of PDGF‐BB/PDGFR‐β to Promote Healing of Superficial Second‐Degree Burn Wounds in Rats,” Journal of Kunming Medical University 42, no. 6 (2021): 7–15, 10.12259/j.issn.2095-610X.S20210639.

[49] V. K. Nguyen , T. T. G. Vu , and S. Homma , “Overview of Methods to Enhance the Oral Bioavailability of Saponins in *Panax notoginseng* (Burk.) F. H. Chen,” VNU Journal of Science: Medical and Pharmaceutical Sciences 40, no. 3 (2024), 10.25073/2588-1132/vnumps.4683.

[50] I. De Luca , P. Pedram , A. Moeini , et al., “Nanotechnology Development for Formulating Essential Oils in Wound Dressing Materials to Promote the Wound‐Healing Process: A Review,” Applied Sciences 11, no. 4 (2021): 1713, 10.3390/app11041713.

[51] G. W. Zhao , X. L. Chen , Z. G. Liao , et al., “Study on the Properties of Long‐Circulating Nanoparticles Loaded With *Panax notoginseng* Saponins,” Chinese Traditional Patent Medicine 33, no. 10 (2011): 1700–1704.

[52] P. F. Xu , R. Zhang , Z. Y. Guan , et al., “Preparation and Intestinal Absorption Characteristics of Chitosan Nanoparticles Loaded With *Panax notoginseng* Saponins,” China Journal of Chinese Materia Medica 47, no. 1 (2022): 95–102, 10.19540/j.cnki.cjcmm.20210412.302. 35178915

[53] Z. Xie , P. Xu , P. P. Zi , et al., “Effects of Low Molecular Weight Chitosan and Its Nanoparticles on Intestinal Permeability of *Panax notoginseng* Saponins,” Chinese Herbal Medicines 51, no. 13 (2020): 3424–3432, 10.7501/j.issn.0253-2670.2020.13.009.

[54] J. Li , L. Li , Y. Yu , et al., “Effect of Carboxymethyl Chitosan‐Sodium Alginate Hydrogel Loaded With Astragalus Membranaceus‐*Panax notoginseng* on Wound Healing in Rats,” Frontiers in Pharmacology 16 (2025): 1526828, 10.3389/fphar.2025.1526828. PMC11813797 39944622

[55] W. J. Tao , “Research Progress of Chitosan Hydrogel for Local Drug Controlled Release,” Chinese Journal of Pharmaceutical Biotechnology 5, no. 4 (2010): 293–296, 10.3969/cmba.j.issn.1673-713X.2010.04.012.

[56] Y. Zuo , G. L. Liu , X. B. Zhong , and X. Q. Huang , “Experimental Study on the Efficacy of *Panax notoginseng*, Coptis Chinensis and Drynaria Rhizome Gel in Promoting the Regeneration of Periodontal Bone Defects,” Journal of Chongqing Medical University 38, no. 9 (2013): 997–1001, 10.11699/cyxb20130909.

[57] Y. Chen , X. Wang , S. Tao , et al., “Research Advances in Smart Responsive‐Hydrogel Dressings With Potential Clinical Diabetic Wound Healing Properties,” Military Medical Research 10, no. 1 (2023): 37, 10.1186/s40779-023-00473-9. PMC10463485 37608335

[58] J. Zhao , L. Chen , A. Ma , et al., “Recent Advances in Coaxial Electrospun Nanofibers for Wound Healing,” Materials Today Bio 29 (2024): 101309, 10.1016/j.mtbio.2024.101309. PMC11570975 39558931

[59] J. Fu , D. Wang , Z. Tang , et al., “NIR‐Responsive Electrospun Nanofiber Dressing Promotes Diabetic‐Infected Wound Healing With Programmed Combined Temperature‐Coordinated Photothermal Therapy,” Journal of Nanobiotechnology 22, no. 1 (2024): 384, 10.1186/s12951-024-02621-2. PMC11218286 38951903

[60] W. Zhang , J. Hu , H. Wu , X. F. Lin , and L. M. Cai , “Stimuli‐Responsive Hydrogel Dressing for Wound Healing,” APL Materials 13, no. 1 (2025): 10601, 10.1063/5.0245545.

[61] Q. Zhou and Y. Liu , “Application of Physiologically Responsive Antimicrobial Materials in Wound Repair,” Materials Science 13, no. 4 (2023): 306–317, 10.12677/MS.2023.134035.

[62] S. S. Chen , J. Wang , and H. S. Zheng , “Determination of the Content of Saponin Components in Rat Skin After Spray Administration of *Panax notoginseng* Saponins by Ultra‐High Performance Liquid Chromatography,” Herald of Medicine 42, no. 2 (2023): 155–159, 10.3870/j.issn.1004-0781.2023.02.003.

[63] Expert Consensus Group on Perioperative Holistic Skincare Clinical Practice of Non‐Invasive Energy Source Aesthetic Medicine in China , “Expert Consensus on Perioperative Holistic Skincare Clinical Practice of Non‐Invasive Energy Source Aesthetic Medicine Projects in China,” Journal of Clinical Dermatology 54, no. 7 (2025): 432–439, 10.16761/j.cnki.1000-4963.2025.07.012.

[64] M. Ding , S. N. Li , C. F. Dong , and S. H. Liu , “Preparation and Characterization of *Panax notoginseng* Saponins‐Chitosan Composite Gel,” Journal of Cangzhou Normal University 38, no. 1 (2022): 14–18, 10.13834/j.cnki.czsfxyxb.2022.01.025.

[65] M. X. Ji and M. Y. Chen , “Effect of Self‐Assembled LL‐37 Antimicrobial Peptide Nanomicelles Prepared by Genetic Engineering Technology on Sepsis‐Induced Acute Lung Injury,” Chinese Journal of Modern Applied Pharmacy 42, no. 17 (2025): 3073–3080, 10.13748/j.cnki.issn1007-7693.20242405.

[66] K. Woo , “The Chronic Wound‐Related Pain Model: Holistic Assessment and Person‐Centered Treatment,” Clinics in Geriatric Medicine 40, no. 3 (2024): 501–514, 10.1016/j.cger.2023.12.013. 38960540

[67] X. C. Cheng , W. Z. Tong , W. Rui , Z. Feng , H. Shuai , and W. Zhe , “Single‐Cell Sequencing Technology in Skin Wound Healing,” Burns & Trauma 12 (2024): tkae043, 10.1093/burnst/tkae043. PMC11497848 39445224

[68] Z. Y. Qian , L. J. Shen , F. Wang , and Y. H. Ruan , “Protective Effect of *Panax notoginseng* Saponins Pretreatment on Rat Intestinal Ischemia‐Reperfusion Injury,” World Chinese Journal of Digestology 20, no. 14 (2012): 1178–1183, 10.11569/wcjd.v20.i14.1178.

[69] X. Liu , J. Chen , L. Zou , et al., “Acupuncture Accelerates Wound Healing via CGRP‐RAMP1‐TSP1‐Mediated Macrophage M2 Polarization,” Chinese Medicine 20, no. 1 (2025): 192, 10.1186/s13020-025-01255-2. PMC12625001 41250153

[70] J. Du and X. L. Liu , “Research Progress of 3D Bioprinting Technology in Skin Wound Repair,” Chinese Journal of Traumatology 30, no. 10 (2014): 1063–1066, 10.3760/cma.j.issn.1001-8050.2014.10.022.

[71] T. Zhu , Y. Feng , J. Xiao , and B. Sun , “Effects of Notoginsenoside R1 on Cutaneous Wound Healing in Mice Through Different Ways of Administration,” Chinese Pharmacological Bulletin 34, no. 3 (2018): 325–330, 10.3969/j.issn.1001-1978.2018.03.007.

[72] D. Y. Zhu , Z. P. Chen , Z. P. Hong , et al., “Injectable Thermo‐Sensitive and Wide‐Crack Self‐Healing Hydrogel Loaded With Antibacterial Anti‐Inflammatory Dipotassium Glycyrrhizate for Full‐Thickness Skin Wound Repair,” Acta Biomaterialia 143 (2022): 203–215, 10.1016/j.actbio.2022.02.041. 35245682

[73] L. Mazini , L. Rochette , B. Admou , S. Amal , and G. Malka , “Hopes and Limits of Adipose‐Derived Stem Cells (ADSCs) and Mesenchymal Stem Cells (MSCs) in Wound Healing,” International Journal of Molecular Sciences 21, no. 4 (2020): 1306, 10.3390/ijms21041306. PMC7072889 32075181

